# Isolation and Characterization of Secondary Metabolites from Endemic and Edible *Polygonum sivasicum* with In Vitro Antioxidant and Cytotoxic Activities

**DOI:** 10.1021/acsomega.5c00438

**Published:** 2025-02-26

**Authors:** Humeyra Karakas, Zeynep Cagman, Cagla Kizilarslan-Hancer, Ebru Erol

**Affiliations:** † Department of Pharmacognosy and Natural Products Chemistry, Health Sciences Institute, Bezmialem Vakif University, 34093 Istanbul, Turkey; ‡ Department of Biochemistry, Faculty of Pharmacy, Bezmialem Vakif University, 34093 Istanbul, Turkey; § Department of Pharmaceutical Botany, Faculty of Pharmacy, Bezmialem Vakif University, 34093 Istanbul, Turkey; ∥ Department of Analytical Chemistry, Faculty of Pharmacy, Bezmialem Vakif University, 34093 Istanbul, Turkey

## Abstract

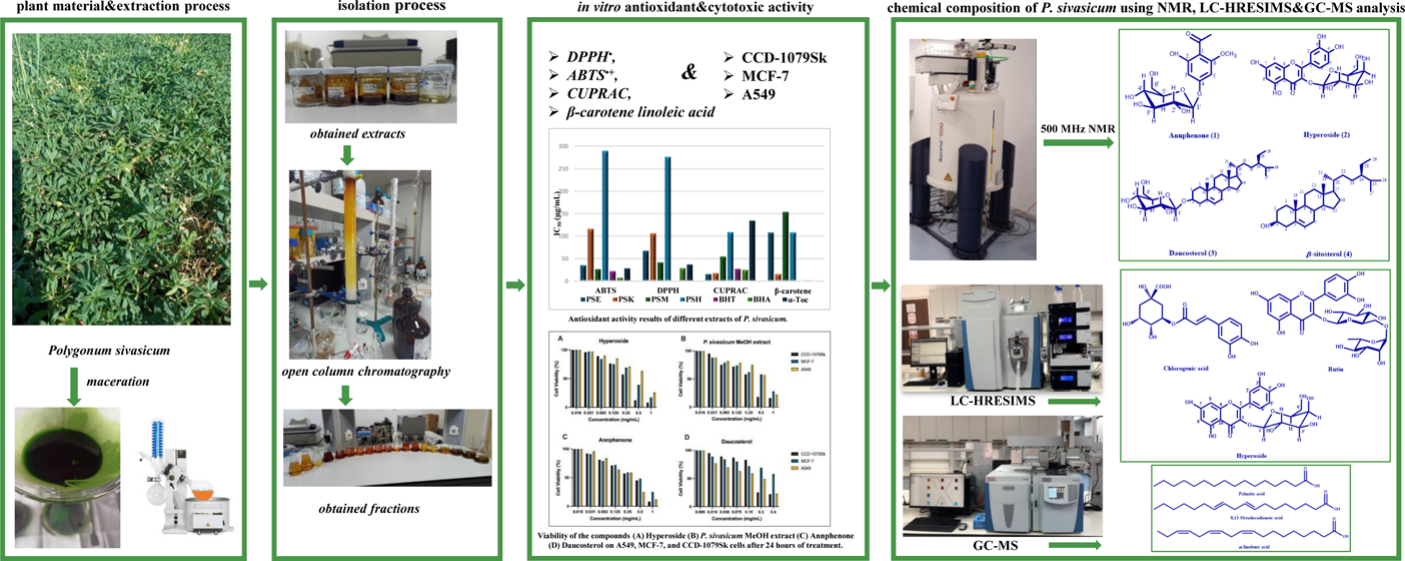

*Polygonum sivasicum* Kit Tan and Yildiz, one of the eight endemic *Polygonum* species in Türkiye, belongs to the Polygonaceae family. Preliminary phytochemical investigation of methanol and hexane extracts of *P. sivasicum* resulted in four compounds, namely, annphenone (**1**), hyperoside (**2**), daucosterol (**3**), and β-sitosterol (**4**). Their structures were elucidated by 1D-, 2D-NMR, and HRESIMS analyses. This study signifies the first isolation of annphenone from the *Polygonum* genus. Antioxidant capabilities of different extracts of *P. sivasicum* were carried out using DPPH^·^, ABTS^·+^, CUPRAC, metal chelating, and β-carotene linoleic acid bleaching assays, and their effectiveness was quantified through IC_50_ values. Furthermore, 27 phenolic compounds were identified using LC-HRESIMS from methanol extract, which has the highest antioxidant activity among the *P. sivasicum* extracts. The major phenolic constituents identified were hyperoside (4535.0 μg/g extract), rutin (4387.4 μg/g extract), and chlorogenic acid (3306.6 μg/g extract). GC–MS analysis determined palmitic acid, α-linolenic acid, and 8,11-octadecadieonic acid as major fatty acids in the hexane extract. The cell viability profile of *P. sivasicum* methanol extract and its isolates hyperoside, annphenone, and daucosterol was evaluated on fibroblast (CCD-1079Sk), breast carcinoma (MCF-7) and lung carcinoma (A549) cell lines. Annphenone exhibited IC_50_ values of 0.25 ± 0.01 mg/mL against the A549 cell line and 0.36 ± 0.02 mg/mL against the MCF-7 cell line. The selective cytotoxicity observed for daucosterol against the A549 cell line, with a high selectivity index of 1.44, underscores its potential as a promising candidate for drug development. The study establishes a framework integrating phytochemical profiling with biological assays to identify therapeutic agents from endemic plants.

## 1 Introduction

*Polygonum* species belong to the Polygonaceae family and consist of annual, perennial, or suffrutescent herbs, or climber plants.^1^ They grow naturally in northern temperate regions.^2^ Being a well-respected plant in Traditional Chinese Medicine (TCM) and Turkish Folk Medicine, roots as well as the aerial parts of *Polygonum* species have been investigated in numerous phytochemical studies^3^ due to their antidiabetic,^4^ antiaging,^5^ hair darkening,^6^ hepatoprotective,^7^ kidney toning,^8^ anti-inflammatory, and diuretic activities.^9^

The studies on this genus were found to be mostly focused on species, such as **P. cuspidatum** Sieb. et Zucc.,^10^*P. multiflorum* Thunb.,^11^*P. aviculare* L.^12^ and *P. cognatum* Meisn.^13^ Anthraquinones, such as emodin, have strong antidiabetic potential;^7^ stilbenes, namely resveratrol, which is acknowledged for its cardioprotective and anticancer properties;^5^ and flavonoids, such as quercetin and rutin, that are known for their powerful antioxidant activities were reported as the main active constituents from *Polygonum* species.^14^

Flavonoids are believed to play a vital role in preventing, delaying, or eliminating oxidative stress-related health conditions such as cardiac problems and neurological diseases due to their hydrogen-donating functional groups, the presence of aromatic rings to stabilize electrons, and ability to form metal chelation.^14^ Nonflavonoid phenolic compounds, such as tannins,^15^ phenolic acids,^16^ and acetophenones,^17^ also contribute significantly to the therapeutic effects of plants. In this regard, natural product discovery has been proven to be an indispensable component of drug development by offering unique and renewable supplies for pharmaceutical research.^18^ Particularly, new drugs targeting lung cancer are found to be urgent as the treatment offered is quite limited. Moreover, it is the most common cancer worldwide and the leading cause of cancer-related deaths among men in Türkiye.^19^ Although breast cancer is less fatal than lung cancer; it is still the most prevalent cancer among women globally.^20^ More than 2.26 million new cases of breast cancer were reported in 2020 with the highest occurring rates observed in countries that have gone through economic transition.^21^

Several species belonging to the *Polygonum* genus have been proven to exhibit anticancer potential, which was also attributed to their well-known stilbenes, anthraquinones, and flavonoids. Based on in vitro and mouse-model studies, **P. cuspidatum** was demonstrated to be effective against osteosarcoma, a type of bone malignancy prevalent among children and adults. Drug target screening and enrichment analyses revealed that seven major metabolites in this plant; namely quercetin, resveratrol, polydatin, emodin, apigenin, catechin and rhein overlap with the drug target diseases, indicating a possible synergistic effect.^22^ In another study, the antiproliferative effect of the supercritical fluid extract of **P. cuspidatum** on human skin melanoma cells, A-375 and A375-S2, was attributed to the extract’s radical scavenging and metal chelating capacity.^23^ The dose-dependent inhibition of cell viability by 70% methanol extract of *P. aviculare* on MCF-7 breast cancer cell lines resulted in 50% cell death at 300 ng/μL and 97% cell death at 400 ng/μL after 24 h.^24^*P. cognatum* was assessed for its antitumor potential against glioblastoma multiforme, an aggressive brain tumor. Use of the methanol extract of *P. cognatum* along with a common anticancer drug, doxorubicin, increased the efficacy of the drug. This result gives hope to potential use of herbal extracts along with anticancer drugs to increase the efficacy while decreasing toxicity by administering low concentrations.^25^

*Polygonum sivasicum* Kit Tan & Yildiz, which is one of the eight endemic *Polygonum* species in Türkiye, is consumed as food locally and named “Madımak, Sivas Madımağı,”^26^ along with other species like *P. cognatum*. It is a suffrutescent perennial plant with a hard woody stock. It is easily distinguished by its short, prostrate stems, and its flowers are sessile or rarely with a pedicel no more than 1.5 mm long.^27,28^ Despite the number of endemic plants present, the lack of phytochemical studies on these plants needs to be addressed. Herein, the phytochemical investigation of *P. sivasicum* is reported for the first time. Therefore, the antioxidant potential of the obtained extracts, namely, hexane, chloroform, ethyl acetate, and methanol extracts together with plant’s water infusion and cooked sample was assessed by using in vitro assays. In addition, the isolation and elucidation of secondary metabolites from the most active methanol extract, including annphenone (**1**, nonflavonoid phenolic), hyperoside (**2**, flavonoid), daucosterol (**3**, steroidal glycoside), and β-sitosterol (**4**, steroid) from the hexane extract were investigated. Also, the phenolic profiling of the polar extract of *P. sivasicum* and fatty acid content of nonpolar extract of *P. sivasicum* were analyzed by using LC-HRESIMS and GC–MS, respectively. The cytotoxic activity of the methanol extract of *P. sivasicum*, and its isolates, annphenone, hyperoside, and daucosterol, was also investigated against the breast cancer MCF-7, lung carcinoma (A549) and human fibroblast (CCD-1079Sk) cell lines.

## 2 Results and Discussion

### 2.1 Isolation of Secondary Metabolites

**Figure 1:**
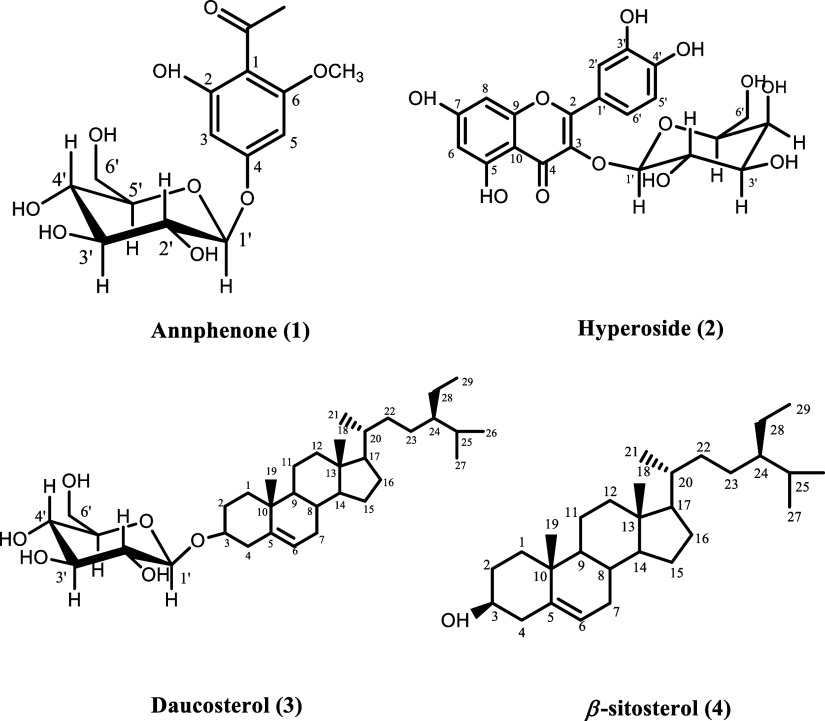
Chemical structures of isolates (**1–4**) from *P. sivasicum*.

The first study on the isolation of the secondary metabolites of *P. sivasicum* led to the identification of four known compounds, namely, annphenone (**1**), hyperoside (**2**), daucosterol (**3**), and β-sitosterol (**4**) (Figure 1). Among these metabolites, annphenone, an acetophenone derivative, was isolated from a limited number of genera in the plant kingdom, namely *Artemisia, Prunus,* and *Euphorbia*. Moreover, isolation of only a few acetophenone glycosides has been reported from *Polygonum* plants so far^29^ and yet this is the first time annphenone was obtained as a pure compound from this genus.

On the other hand, flavonoids and their glycosides were widely observed in *Polygonum* genus. Hyperoside, a flavonoid glycoside, was first isolated from *Hypericum perforatum* in 1937.^30^ Its isolation from several *Polygonum* species, such as *P. multiflorum*,^31^*P. nepalense*,^32^ and *P. alpinum*,^33^ and *P. salicifolium*(34) has been reported since then. Although not as common, daucosterol, a β-sitosterol glycoside, was obtained from *P. multiflorum*,^35^*P. hydropiper*^36^ and *P. polystachyum*(37) as a pure substance as well. β-sitosterol was found in numerous species of this genus, namely in **P. cuspidatum*,*(38)*P. maritimum*,^39^*P. hydropiper*,^40^*P. bistorta*,^41^ and *P. polystachyum.*(37) Herein, the isolation of annphenone, hyperoside, daucosterol, and β-sitosterol from *P. sivasicum* is reported for the first time.

Compound **1** was isolated as a white, amorphous powder. The molecular formula was determined as C_15_H_20_O_9_ based on the HRESIMS (Figure S6) peaks observed at [M+H]^+^*m*/*z* 345.11752 (calcd for C_15_H_20_O_9_, 344.11073) and a sodium adduct ion peak [M+Na]^+^ at *m*/*z* 367.09949 (calcd 367.10050). In ^1^H NMR spectrum (CD_3_OD, 500 MHz) of compound **1** two aromatic protons δ 6.33 (1H, d, *J* = 2.4 Hz, H-5) and 6.13 (1H, d, *J* = 2.4 Hz, H-3) detected to be in resonance (Figure S1). Their coupling constants (*J* = 2.4 Hz) indicated that these two protons are in meta positions.^42^ Two singlets detected at 2.71 (3H, s) and δ 3.81 (3H, s) are attributed to methyl linked to an acetyl group and methoxy, respectively. The investigation of the ^13^C NMR spectrum (CD_3_OD, 125 MHz) revealed not only the presence of a carbonyl group supported by a quaternary carbon signal at δ 205.66; but also, the existence of three carbons signals in the aromatic region with chemical shifts at δ 168.17, 168.08 and 162.76, possibly connected to oxygen-bearing groups (Figure S2). A total of 15 carbons were resonated in the ^13^C NMR. In HRESIMS of compound **1**, a peak at *m*/*z* 183.06505 is considered evidence of the existence of an acetophenone backbone after glucose cleavage (Figure S6).

**Figure 2:**
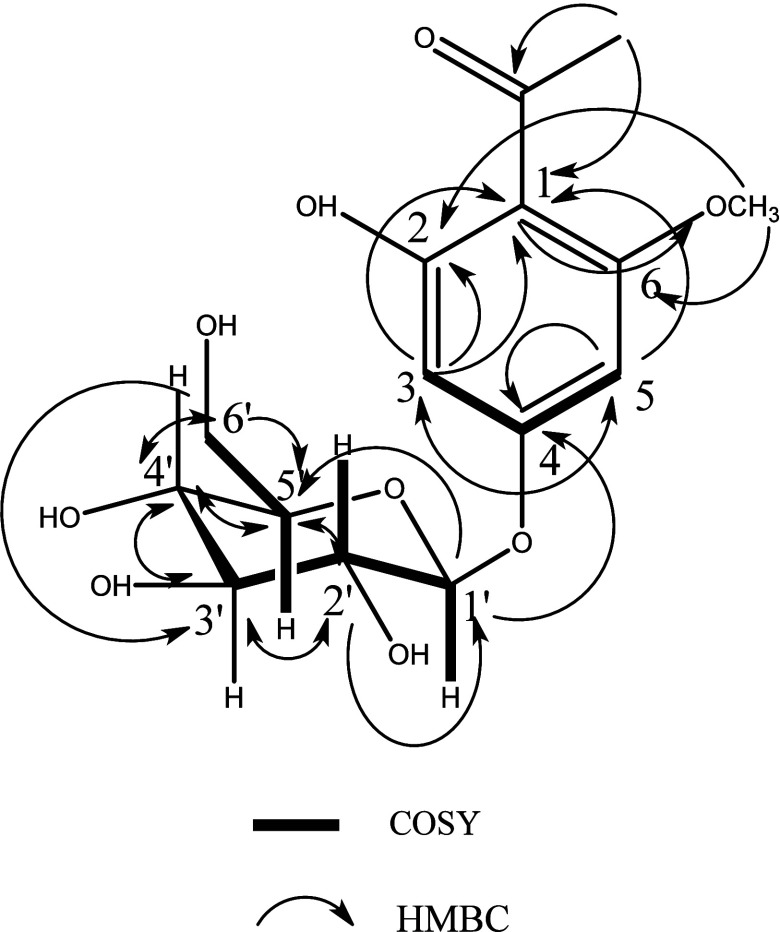
HMBC and COSY correlations of compound (**1**).

Furthermore, HMBC correlations supported that the structure is acetophenone glycoside and that the anomeric proton is connected to the aromatic ring at C-4 (Figure S4). All the correlations detected in HMBC and COSY spectrum were listed in (Table 1) and depicted in (Figure 2). Spectral data were compared to the literature data^43^ to confirm compound **1** as 2,4-dihydroxy-6-methoxy-acetophenone 4-*O*-β-D-glucopyranoside.

**Table 1:** ^13^C-NMR (CD_3_OD, 125 MHz), ^1^H-NMR (CD_3_OD, 500 MHz), HMBC, and COSY Correlations of Annphenone (**1**)

pozisyon	δ_c_	δ_H_	HMBC (H → C)	COSY
1	107.99			
2	168.17			
3	96.90	6.13 (1H, d, *J* = 2.4 Hz)	C-1; C-2; C-5	H-5
4	162.76			
5	95.23	6.33 (1H, d, *J* = 2.4 Hz)	C-1; C-3; C-4	H-3
6	168.08			
1′	102.62	5.08 (1H, d, *J* = 7.7 Hz)	C-4; C-5′	H-2′
2′	75.27	3.54(1H, dd, *J* = 9.2; 7.7 Hz)	C-3′/ C-5′; C-1′	H-1′
3′	79.00	3.47 (1H, m)	C-2′; C-4′	
4′	71.73	3.38 (1H, m)	C-6′; C-3′/ C-5′	
5′	79.01	3.47 (1H, m)	C-4′; C-2′	H-6a′, H-6b′
6a′	62.96	3.71 (1H, dd, *J* = 12.2; 6.0 Hz)	C-3′/ C-5′	H-5′, H-6b′
6b′		3.91 (1H, dd, *J* = 12.1; 2.3 Hz)	C-4′	H-5′, H-6a′
C=O	205.66			
–OCH_3_	56.65	3.81 (3H, s)	C-2; C-6	
–CH_3_	34.12	2.71 (3H, s)	C-1; C=O	

The investigation of the ^13^C NMR (DMSO-*d*_6_, 125 MHz) spectrum of compound **2** revealed the presence of a carbonyl group (δ 178.00) and a sugar moiety due to 5 signals in 60–77 ppm range and an anomeric carbon signal at δ 102.26 (Figure S8). Four aromatic carbons with possible oxygen-bearing groups attached were observed further downfield at δ 156.73, 156.80, 161.74, and 164.62. When evaluated in conjunction with ^1^H NMR spectrum (DMSO-*d*_6_, 500 MHz) peaks monitored at 9.15, 9.73, 10.86, and 12.64 ppm, the structure was determined to contain four hydroxyl groups (Figure S7). Furthermore, HMBC correlations (Figure S10) indicated that anomeric proton is attached to aromatic carbon at C-3 and the sugar moiety was found to be galactose in accordance with the previous literature data.^44,45^ In conclusion, compound **2** was identified to be hyperoside.

In the ^1^H NMR spectrum (DMSO-*d*_6_, 500 MHz) of compound **3**, observed signals at δ 0.65 (3H, s), 0.90 (3H, d*, J* = 6.5 Hz), 0.81 (9H, m), and 0.95 (3H, s) for six methyl groups in addition to an olefinic proton signal at 5.32 ppm (1H, dt, *J* = 4.7, 2.0 Hz) were attributed to the characteristic peaks of steroids (Figure S11). The seven proton signals in the 2.88–4.21 ppm range in the ^1^H NMR spectrum and six carbon signals 61.10, 70.11, 73.48, 76.78 (2 C), and 100.77 ppm in the ^13^C NMR spectrum indicate the presence of a sugar moiety (Figure S13). Moreover, coupling constants of H-1′ (d, *J* = 7.8 Hz) and H-2′ (t, *J* = 8.4 Hz) suggest a sugar in β-position. In comparison of 2D-NMR data (Figures S13–S15) of compound **3** with the literature data,^46^ compound **3** was found to be β-sitosterol-3-*O*-β-glucopyranoside.

Based on ^1^H NMR (CDCl_3_, 500 MHz) spectral data (Figure S16) comparison of compound **4** with previously isolated β-sitosterol from a green alga (Figures S17 and S18), *Caulerpa cylindracea*,^47^ the structure was elucidated as β-sitosterol. ^13^C NMR (CDCl_3_, 125 MHz) data (Figure S19) were further compared with another literature for final confirmation as well.^48^

### 2.2 Structural Identification

#### 2.2.1 Annphenone: 2,4-dihydroxy-6-methoxy-acetophenone 4-*O*-β-D-glucopyranoside (**1**)

White amorphous powder. ^1^H NMR (500 MHz, CD_3_OD): δ 6.33 (1H, d, *J* = 2.4 Hz, H-5), 6.13 (1H, d, *J* = 2.4 Hz, H-3), 5.08 (1H, d, *J* = 7.7 Hz, H-1′), 3.91 (1H, dd, *J* = 12.1; 2.3 Hz, H-6b′), 3.81 (3H, s, −OCH_3_), 3.71 (1H, dd, *J* = 12.2; 6.0 Hz, H-6a′), 3.54 (1H, dd, *J* = 9.2; 7.7 Hz, H-2′), 3.47 (2H, m, H-3′ and H-5′), 3.38 (1H, m, H-4′), 2.71 (3H, s, −CH_3_). ^13^C NMR (125 MHz, CD_3_OD): δ 205.66 (C=O), 168.17 (C-2), 168.08 (C-6), 162.76 (C-4), 107.99 (C-1), 102.62 (C-1′), 96.90 (C-3), 95.23 (C-5), 79.01 (C-5′), 79.00 (C-3′), 75.27 (C-2′), 71.73 (C-4′), 62.96 (C-6′), 56.65 (−OCH_3_), 34.12 (−CH_3_).

#### 2.2.2 Hyperoside: Quercetin-3-*O*-β-galactopyranoside (**2**)

Bright yellow, amorphous powder. ^1^H NMR (500 MHz, DMSO-*d*_6_): δ 12.64 (−OH), 10.86 (−OH), 9.73 (−OH), 9.15 (−OH), 7.67 (1H, dd, *J* = 8.5, 2.2 Hz, H-6′), 7.52 (1H, d, *J* = 2.3 Hz, H-2′), 6.83 (1H, d, *J* = 8.5 Hz, H-5′), 6.41 (1H, d, *J* = 2.1 Hz, H-8), 6.21 (1H, d, *J* = 2.1 Hz, H-6), 5.36 (1H, d, *J* = 7.7 Hz, H-1″), 3.64 (1H, t, *J* = 3.9 Hz, H-4″), 3.57 (1H, ddd, *J* = 9.6, 7.7, 4.5 Hz, H-2″), 3.46 (1H, m, H-6a″), 3.37 (1H, H-3″), 3.32 (1H, H-5″), 3.29 (1H, m, H-6b″). ^13^C NMR (125 MHz, DMSO-*d*_6_): δ 178.00 (C-4), 164.62 (C-7), 161.74 (C-5), 156.80 (C-2), 156.73 (C-9), 148.97 (C-4′), 145.34 (C-3′), 133.97 (C-3), 122.52 (C-6′), 121.59 (C-1′), 116.42 (C-2′), 115.68 (C-5′), 104.42 (C-10), 102.26 (C-1″), 99.16 (C-6), 94.00 (C-8), 76.35 (C-5″), 73.67 (C-3″), 71.69 (C-2″), 68.42 (C-4″), 60.63 (C-6″).

#### 2.2.3 Daucosterol: β-sitosterol-3-*O*-β-glucopyranoside (**3**)

White amorphous solid. ^1^H NMR (500 MHz, DMSO-*d*_6_): δ 5.32 (1H, dt, *J* = 4.7, 2.0 Hz, H-6), 4.21 (1H, d, *J* = 7.8 Hz, H-1′), 3.64 (1H, d, *J* = 11.5 Hz, H-6a′), 3.46 (1H, tt, *J* = 11.4, 6.9, 4.5 Hz, H-3), 3.38 (1H, m, H-6b′), 3.11 (1H, t, *J* = 8.8 Hz, H-3′), 3.06 (1H, ddd, *J* = 9.7, 5.9, 2.1 Hz, H-5′), 3.01 (1H, t, *J* = 9.2 Hz, H-4′), 2.88 (1H, t, *J* = 8.4 Hz, H-2′), 2.12/2.37 (2H, m, H-20), 1.14/1.95 (2H, m, H-4), 1.50/1.92 (2H, m, H-7), 1.48/1.80 (2H, m, H-12), 0.99/1.79 (2H, m, H-1), 1.22/1.78 (2H, m, H-16), 1.62 (1H, m, H-25), 1.03/1.53 (2H, m, H-15), 1.40/1.47 (2H, m, H-11), 1.39 (1H, m, H-8), 1.34 (2H, m, H-22), 1.14/1.32 (2H, m, H-2), 1.22 (2H, m, H-28), 1.16 (2H, m, H-23), 1.08 (1H, m, H-17), 0.98 (1H, m, H-14), 0.95 (3H, s, H-19), 0.90 (3H, d, *J* = 6.5 Hz, H-21), 0.90 (1H, m, H-24), 0.89 (1H, m, H-9), 0.81 (3H, m, H-26), 0.81 (3H, m, H-27), 0.81 (3H, m, H-29), 0.65 (3H, s, H-18). ^13^C NMR (125 MHz, DMSO-*d*_6_): δ 140.46 (C-5), 121.26 (C-6), 100.77 (C-1′), 76.89 (C-3), 76.78 (C-3′), 76.78 (C-5′), 73.48 (C-2′), 70.11 (C-4′), 61.10 (C6′), 56.19 (C-14), 55.43 (C-17), 49.61(C-9), 45.14 (C-24), 41.87 (C-13), 40.11 (C-4), 38.31 (C-20), 36.84 (C-1), 36.23 (C-10), 35.50 (C-22), 33.35 (C-2), 31.44 (C-8), 31.39 (C-7), 29.28 (C-12), 28.70 (C-25), 27.82 (C-16), 25.41 (C-23), 23.89 (C-15), 22.61 (C-28), 20.61 (C-11), 19.75 (C-26), 19.13 (C-19), 18.95 (C-27), 18.64 (C-21), 11.81 (C-29), 11.70 (C-18).

#### 2.2.4 β-sitosterol (**4**)

Needle-like crystals. ^1^H NMR (500 MHz, CDCl_3_): δ 5.35 (1H, dd, *J* = 5.1, 2.4 Hz, H-6), 3.52 (1H, tt, *J* = 11.1, 4.6 Hz, H-3), 2.20–2.31 (2H, m, H-20), 2.00 (2H, m, H-4), 1.55/1.95 (2H, m, H-7), 0.98/1.84 (2H, m, H-1), 1.86 (2H, m, H-12), 1.23/1.86 (2H, m, H-16), 1.66 (1H, m, H-25), 1.55 (2H, m, H-15), 1.50 (2H, m, H-11), 1.36 (1H, m, H-8), 1.33 (2H, m, H-22), 1.29 (2H, m, H-2), 1.25 (2H, m, H-28), 1.18 (2H, m, H-23), 1.08 (1H, m, H-17), 1.01 (3H, s, H-19), 0.98 (1H, m, H-14), 0.92 (3H, d, *J* = 6.6 Hz, H-21), 0.89 (1H, m, H-24), 0.86 (1H, m, H-9), 0.83 (3H, m, H-26), 0.83 (3H, m, H-27), 0.83 (3H, m, H-29), 0.68 (3H, s, H-18). ^13^C NMR (125 MHz, CDCl_3_): δ 37.26 (C-1), 33.95 (C-2), 71.83 (C-3), 39.78 (C-4), 140.77 (C-5), 121.75 (C-6), 31.67 (C-7), 31.93 (C-8), 50.13 (C-9), 36.52 (C-10), 21.10 (C-11), 29.72 (C-12), 42.33 (C-13), 56.77 (C-14), 24.32 (C-15), 28.27 (C-16), 56.05 (C-17), 11.88 (C-18), 19.42 (C-19), 42.31 (C-20), 18.79 (C-21), 36.16 (C-22), 26.06 (C-23), 45.84 (C-24), 29.14 (C-25), 19.84 (C-26), 19.04 (C-27), 23.07 (C-28), 12.00 (C-29).

### 2.3 Quantification of the Phenolic Compounds by LC-HRESIMS Analysis

**Figure 3:**
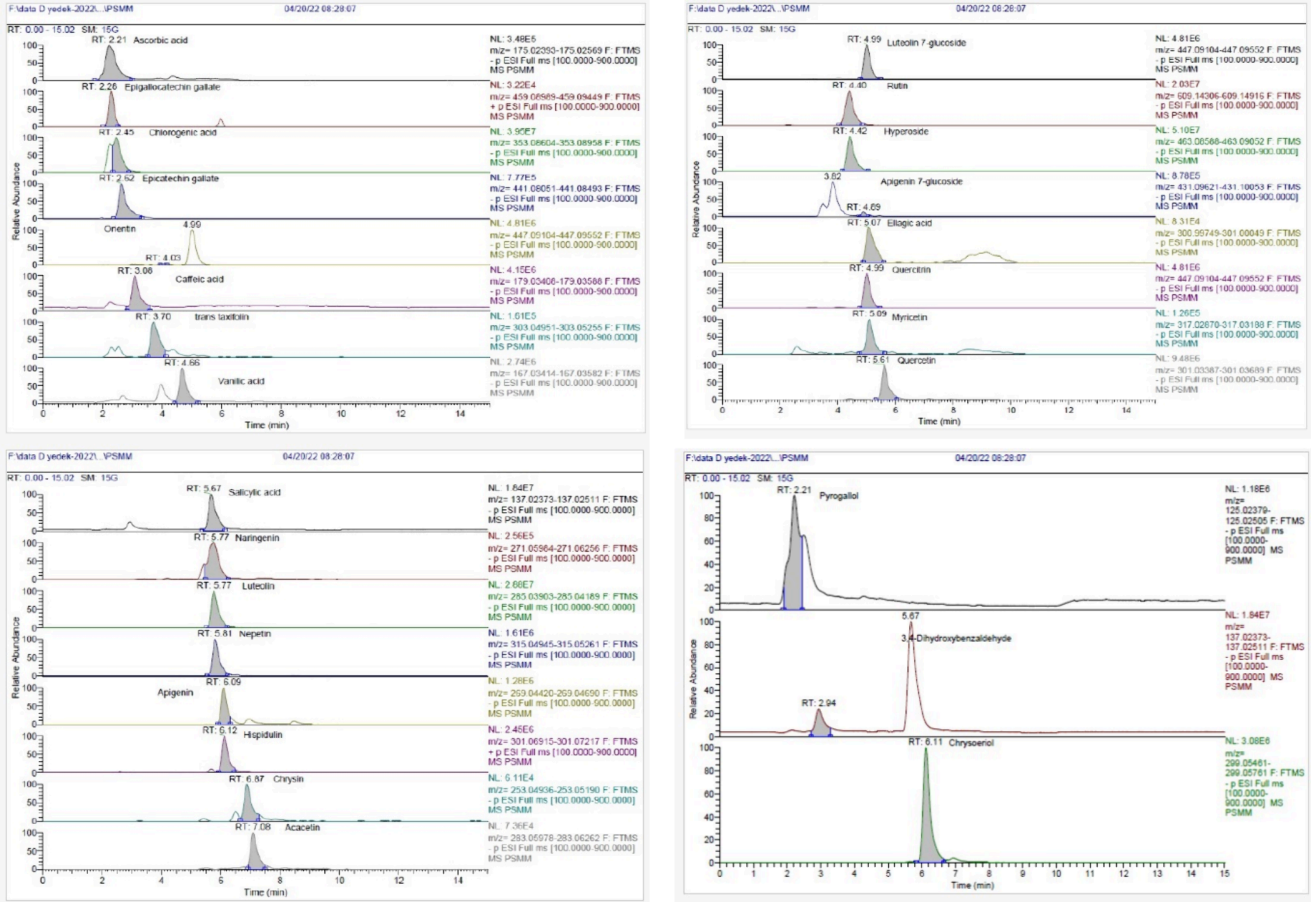
LC-HRESIMS chromatograms of the methanol extract of the *P. sivasicum*.

The methanol extract, which has the highest antioxidant activity among the *P. sivasicum* extracts, was evaluated for phenolic profiling using LC-HRESIMS against 60 standards (Table S2). This study can be considered as a precursor for future isolation studies in this species as the presence of these phenolics or lack thereof implies screening some of the most common standards, mostly phenolics, found in plants. LC-HRESIMS analysis resulted in the identification of 27 phenolic compounds (Table 2, Figure 3). Hyperoside (4535.0 μg/g extract), rutin (4387.4 μg/g extract), and chlorogenic acid (3306.6 μg/g extract) were quantified at the highest concentrations.

**Table 2:** Quantified Phenolics in the Methanol Extract of *P. sivasicum* by Using LC-HRESIMS

phenolics	molecular formula	*t*_R_^a^ (min)	(μg/g extract)
ascorbic acid	C_6_H_8_O_6_	1.99	104.40
(-)-epigallocatechin gallate	C_22_H_18_O_11_	2.21	7.60
chlorogenic acid	C_6_H_18_O_9_	2.43	3306.60
pyrogallol	C_6_H_6_O_3_	2.55	4.60
(-)-epicatechin gallate	C_22_H_18_O_10_	2.69	70.40
orientin	C_21_H_20_O_11_	3.09	12.40
caffeic acid	C_9_H_8_O_4_	3.17	74.80
3,4-dihydroxybenzaldehyde	C_7_H_6_O_3_	3.25	1.40
(+)-*trans* taxifolin	C_15_H_12_O_7_	3.85	4.20
vanillic acid	C_8_H_8_O_4_	4.12	647.20
luteolin 7-glucoside	C_21_H_20_O_11_	4.24	9.20
rutin	C_27_H_30_O_16_	4.48	4387.40
hyperoside	C_21_H_20_O_12_	4.58	4535.00
apigenin 7-glucoside	C_21_H_20_O_10_	5.00	3.00
ellagic acid	C_14_H_6_O_8_	5.12	9.00
quercitrin	C_15_H_10_O_7_	5.13	151.60
myricetin	C_15_H_10_O_8_	5.15	4.20
quercetin	C_15_H_10_O_7_	5.68	97.80
salicylic acid	C_7_H_6_O_3_	5.72	294.00
naringenin	C_15_H_12_O_5_	5.74	8.40
luteolin	C_15_H_10_O_6_	5.84	144.80
nepetin	C_16_H_12_O_7_	5.86	13.00
chrysoeriol	C_16_H_12_O_6_	6.12	16.60
apigenin	C_15_H_10_O_5_	6.20	6.40
hispidulin	C_16_H_12_O_6_	6.24	50.00
chrysin	C_15_H_10_O_4_	6.99	0.40
acacetin	C_16_H_12_O_5_	7.05	1.00

a *t*_R_: retention time.

Major phenolics identified by LC-HRESIMS were repeatedly linked with a strong antioxidant capacity and anticancer potential. Hyperoside, as the major phenolic found in *P. sivasicum*, has been reported to be the subject of several anticancer studies, such as skin,^49^ ovarian (SKOV-3 and HO-8910),^50^ cervical,^51^ pancreatic (MIA-PaCa-2)^52^ cell lines. Rutin, as one of the major phenolics determined by LC-HRESIMS in the present study, was studied for its antioxidant activity in a 2008 research (total antioxidant activity and reducing power, hydroxyl radical scavenging assay, superoxide radical scavenging assay, DPPH radical scavenging assay, and lipid peroxidation assay).^53^

Rutin, isolated from *Tanacetum alyssifolium* (Bornm.) Grierson, was evaluated for its cytotoxicity against the breast carcinoma cell line (MCF-7)^54^ and the effect of rutin on the viability, superoxide anion production, adhesion, and migration of human lung (A549) and colon (HT29 and Caco-2) cancer cell lines was assessed by Sghaier et al.^55^ Another major phenolic, chlorogenic acid, was evaluated for its cytotoxicity against breast carcinoma (MCF-7 and MAD-MB-231) cell lines and was found to inhibit DNA methylation partially.^56^ In another study, chlorogenic acid was shown to exhibit a significant inhibitory effect on the proliferation of A549 cells both in vitro and in vivo experiments.^57^

### 2.4 Determination of Fatty Acid Content by Using GC–MS

GC–MS analysis of the hexane extract revealed 19 fatty acids (Table 3). and their relative concentrations. While palmitic acid (25.28%) was identified as the major saturated fatty acid, α-linolenic acid (28.49%) and 8,11-octadecadieonic acid (23.23%) were determined as the major unsaturated fatty acids in the sample. The α-linolenic acid (ALA), a vital omega-3 fatty acid, comprises 50–60% of fatty acids in flaxseed and 30% of fatty acids in fish oil.^58^ A high level of ALA in *P. sivasicum* was found to be significant in terms of highlighting the nutritional value of an endemic and edible plant. ALA was also found in *P. equisetiforma* (calcd. %30)^59^ and detected in *P. bistorta*, *P. maritimum*, and *P. orientale*.^60^

**Table 3:** Fatty Acid Composition (%) of *Polygonum sivasicum* Hexane Extract

retention time	compound name	molecular formula	C:D	area %
5.92	pelargonic acid	C_9_H_18_O_2_	9:0	0.05
6.51	capric acid	C_10_H_20_O_2_	10:0	0.11
7.61	lauric acid	C_12_H_24_O_2_	12:0	0.66
7.76	azelaic acid	C_11_H_20_O_4_	9:0	0.12
8.25	tridecylic acid	C_14_H_28_O_2_	13:0	0.03
8.99	myristic acid	C_14_H_28_O_2_	14:0	1.06
9.86	pentadecanoic acid	C_15_H_30_O_2_	15:0	0.40
10.67	palmitoleic acid	C_16_H_30_O_2_	16:1	0.80
10.87	**palmitic acid**	**C**_**16**_**H**_**32**_**O**_**2**_	**16:0**	**25.28**
12.00	heptadecanoic acid	C_17_H_34_O_2_	17:0	0.40
12.90	**8,11-octadecadienoic acid**	**C**_**18**_**H**_**32**_**O**_**2**_	**18:2**	**23.23**
12.99	**α-linolenic acid**	**C**_**18**_**H**_**30**_**O**_**2**_	**18:3**	**28.49**
13.25	stearic acid	C_18_H_36_O_2_	18:0	4.18
16.26	arachidic acid	C_20_H_40_O_2_	20:0	2.10
18.38	heneicosanoic acid	C_21_H_42_O_2_	21:0	0.22
21.16	behenic acid	C_22_H_44_O_2_	22:0	3.33
24.80	tricosanoic acid	C_23_H_46_O_2_	23:0	0.78
28.30	nervonic acid	C_24_H_46_O_2_	24:1	0.11
29.64	lignoceric acid	C_24_H_48_O_2_	24:0	3.54
total				95

### 2.5 In Vitro Antioxidant Activity

The antioxidant activities of six different extracts of *P. sivasicum* for five different assays are shown in Table 4 displaying the IC_50_ value, which represents the concentration of an antioxidant compound needed to decrease the activity of reactive oxygen species (ROS) by 50%. Methanol extract was found to be the most active one in ABTS cation radical assay with 25.9 μg/mL IC_50_ value which was comparable to the standards BHT (21.67 μg/mL) and α-tocopherol (27.70 μg/mL). Based on the DPPH free radical scavenging assay, methanol extracts’ IC_50_ value was measured as 41.26 μg/mL which was also the most active extract and comparable to the IC_50_ value of the standard α-tocopherol (36.35 μg/mL). In metal chelating assay, all extracts showed low activity except the cooked plant sample (155.48 μg/mL). Higher activity of this extract was attributed to the formation of new metal chelating sides due to the cleavage of the glycosidic bonds. A similar result was reported in the literature with cooked black rice sample and the increase was explained with the fact that new phenolics might have formed as a result of decomposition of phenolics due to the heating process.^61^ In the CUPRAC method, ethyl acetate and chloroform extracts were determined as the most active extracts with *A*_0.5_ values 15.21 and 17.39 μg/mL, respectively. These two extracts were proven to be more potent in terms of antioxidant activity compared to the standards BHA (24.49 μg/mL) and BHT (26.85 μg/mL). All extracts, except the chloroform extract, demonstrated medium antioxidant activity in the β-carotene-linoleic acid method, with the chloroform extract exhibiting the highest activity with an IC_50_ value of 14.93 μg/mL.

**Table 4:** Antioxidant Activity Results of Different Extracts of *P. sivasicum* (IC_50_, μg/mL)^a^

	ABTS	DPPH	metal chelating	CUPRAC	β-carotene
	IC_50_ (μg/mL)^b^	IC_50_ (μg/mL)^b^	IC_50_ (μg/mL)^b^	IC_50_ (μg/mL)^b^*^,^*^c^	IC_50_ (μg/mL)^b^
extracts					
PSH	290.00 ± 3.20	276.20 ± 2.20	1418.62 ± 1.12	108.49 ± 6.75	108.14 ± 0.75
PSE	34.80 ± 2.10	67.10 ± 2.30	1331.62 ± 5.34	15.21 ± 2.43	108.00 ± 3.63
PSK	116.30 ± 2.99	106.30 ± 3.17	1314.93 ± 4.12	17.39 ± 2.49	14.93 ± 0.11
PSM	25.90 ± 1.45	41.26 ± 1.01	1018.62 ± 6.64	54.09 ± 3.81	153.77 ± 2.37
PSS	832.9 ± 0.90	746.11 ± 2.9	1031.62 ± 2.34	133.45 ± 2.30	158.69 ± 2.14
PSC	42.60 ± 0.88	113.78 ± 2.61	155.48 ± 0.62	94.33 ± 3.40	772.25 ± 3.70

standards^d^					
BHT	21.67 ± 0.87			26.85 ± 1.50	
BHA	7.23 ± 0.01	28.59 ± 0.06		24.49 ± 0.19	1.34 ± 0.04
α-Toc	27.70 ± 0.28	36.35 ± 0.24		134.53 ± 0.19	
EDTA			26.85 ± 1.50		

a The values represents the mean ± SEM of three parallel measurements (*p* < 0.05).

b IC_50_ values and *A*_0.5_^c^ values represent the means ± SEM of three parallel measurements (*p* < 0.05).

c *A*_0.5_ symbol correspond the μg/mL concentration of 0.500 Absorbance value.

d BHT: Butylated hydroxy toluene; BHA: Butylated hydroxy anisol; α-Toc: Tocopherol; EDTA: Ethylenediaminetetraacetic Acid.

### 2.6 In Vitro Cytotoxic Activity

Annphenone, isolated from a *Euphorbia* species, was shown to exhibit moderate antioxidant activity in DPPH^·^ scavenging assay with IC_50_ value: 23.23 ± 1.8 μg/mL. In the same study, annphenone and six other acetophenone glycoside analogues were shown to demonstrate no cytotoxicity against MCF-7, A549, Hep-3B, U118, and U87 cell lines. However, data related to the experiments were not included in the paper.^62^ Annphenone has a significant antiproliferative effect on HepG2 cells with an IC_50_ value of 2.0 μg/mL which is close to the IC_50_ value of 5-fluorouracil (0.33 μg/mL), a common chemotherapy drug.^17^ Furthermore, the cytoprotective effect of annphenone in V79–4 cells against H_2_O_2_-induced apoptosis was confirmed by a 2008 study.^63^

Hyperoside, detected as one of the major phenolics by LC-HRESIMS, was also isolated during this study. The isolation of hyperoside both from *Helichrysum* and *Polygonum* species was reported previously.^34,64^ Its high therapeutic potential for various diseases such as neurological problems, namely depression and epilepsy; breast, lung, and liver cancers can be attributed to its high antioxidant capacity.^33,34^

In a 2018 study, the cytotoxicity of daucosterol, a steroidal glycoside of β-sitosterol, was assessed on two different breast cancer cell lines, MCF-7 and MDA-MB-231. IC_50_ values of daucosterol on MCF-7 and MDA-MB-231 after 24 h were found to be 30.82 and 49.76 mM, respectively. Moreover, antibreast cancer activity of daucosterol was further supported by evaluating in vivo on mice. Daucosterol demonstrated a decrease in the tumor weight and tumor volume in a dose-dependent manner. Daucosterol’s inhibition activity on MCF-7 cell lines was also related to its phytosterol property as breast cancer cell lines such as MCF-7 are known to be estrogen receptor active.^46^

**Figure 4:**
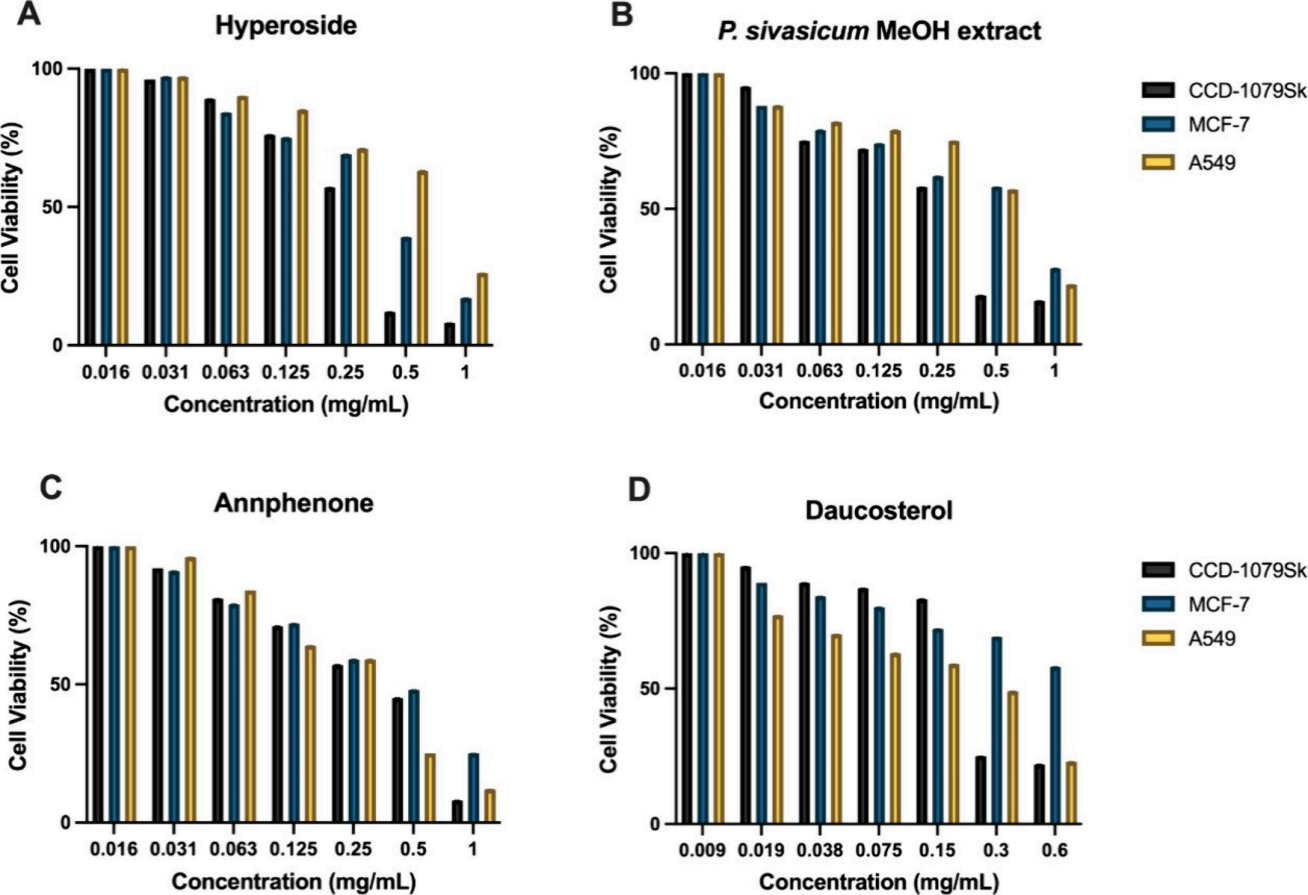
Viability of the compounds (A) Hyperoside, (B) *P. sivasicum* MeOH extract, (C) Annphenone, (D) Daucosterol on A549, MCF-7, and CCD-1079Sk cells after 24 h of treatment.

In consideration of previous literature data on four isolates, cytotoxicity of compounds hyperoside, annphenone, daucosterol, and *P. sivasicum* methanol extract against MCF-7, A549 tumor cells, and CCD nontumor cells was evaluated for 24 h using the MTT assay and IC_50_ values were calculated (Table 5). As shown in Figure 4, a concentration-dependent decrease in the survival of all cells affected by these compounds was observed. However, this decrease caused by hyperoside and *P. sivasicum* methanol extract is greater in noncancerous CCD-1079Sk cells. While these two compounds had the most toxic effect on CCD-1079Sk cells over a 24 h period, they were more toxic to breast cancer cells than the lung cancer cells. Annphenone was more effective in breast cancer cells and showed more cytotoxic effects in both cancer cells than in noncancerous cells. Daucosterol, which was more cytotoxic to lung cells than to noncancerous cells, was less cytotoxic to the viability of breast cancer cells than to the other two cells. Doxorubicin, an anticancer drug, was used as a positive control. When the IC_50_ values of each substance in all three cell lines were evaluated statistically within themselves, a significant difference was found. When the IC_50_ values of hyperoside in all cell lines were compared, the difference between the breast cancer cell line and the noncancerous cell line was not found to be statistically significant (*p* > 0.05), however, in the lung cancer cell line, the IC_50_ value was found to be statistically significant with the IC_50_ value in both the breast cancer cell line and the noncancerous cell line (*p* < 0.001). When the IC_50_ values of *P. sivasicum* MeOH extract in breast cancer and lung cancer cell lines were compared separately with the noncancerous cell line, the difference between the cancerous and noncancerous cell lines was found to be statistically significant (*p* < 0.0001). In addition, the IC_50_ values in both cancerous cells gave a statistically significant difference (*p* < 0.05). The difference between the IC_50_ values of annphenone, which is more cytotoxic in lung cancer cells compared to noncancerous cells, in these cells was not found to be statistically significant. Similarly, the difference between the IC_50_ values in lung cancer cells and noncancerous cells was not statistically significant (*p* > 0.05). Only the difference between the two cancer cells was found to be statistically significant (*p* < 0.05). Like annphenone, the difference between the IC_50_ values of daucosterol, which is more cytotoxic in lung cancer cells than in noncancerous cells, was also found to be statistically significant (*p* < 0.05). In addition, the difference between the IC_50_ values of daucosterol in breast cancer and noncancerous cells was found to be statistically highly significant (*p* < 0.0001) and the difference between the IC_50_ values in both cell lines was found to be statistically highly significant (*p* < 0.0001) (Figure S23).

**Table 5:** Cytotoxic Effect of Isolated Compounds against CCD-1079Sk, MCF-7, and A549 Cell Lines^a^

	IC_50_ (mg/mL), 24 h			selectivity index		
samples	CCD-1079Sk	MCF-7	A549	MCF-7	A549	*p* value
*P. sivasicum* MeOH extract (PSM)	0.24 ± 0.03	0.44 ± 0.01	0.50 ± 0.01	0.54	0.48	<0.0001
Hyperoside	0.25 ± 0.04	0.37 ± 0.02	0.60 ± 0.07	0.67	0.41	0.0003
Annphenone	0.30 ± 0.05	0.36 ± 0.02	0.25 ± 0.01	0.83	1.20	0.0152
Daucosterol	0.26 ± 0.03	0.58 ± 0.02	0.18 ± 0.02	0.44	1.44	<0.0001
Doxorubicin	0.28 ± 0.01	0.04 ± 0.01	0.01 ± 0.01	7.0	28.0	

a Values are mean ± SD (*n* = 3).

## 3 Conclusions

The first phytochemical analyses of an endemic and edible plant *P. sivasicum* resulted in the isolation of four known compounds (**1**–**4**) from hexane and methanol extracts. Among these compounds, annphenone was isolated from a *Polygonum* genus for the first time. Hyperoside, one of the major phenolics quantified by LC-HRESIMS, was also obtained as a pure compound. Antioxidant activity tests revealed that the methanol extract was the most active in ABTS^·+^ and DPPH^·^ assays. On the other hand, ethyl acetate extract was the most potent in CUPRAC assay. While the cooked sample was found to be most active in metal chelating, the chloroform extract was the most active one in the β-carotene test. According to LC-HRESIMS results, 27 phenolic compounds were determined, with chlorogenic acid, hyperoside, and rutin being the most prominent. GC–MS analysis revealed that palmitic acid was the major saturated fatty acid; and α-linolenic acid and 8,11-octadecadieonic acid were the major unsaturated fatty acids in hexane extract. In vitro cytotoxicity results showed that annphenone had a slightly higher toxicity rate in lung cancer (A549) cells than in noncancerous (CCD-79Sk) cells. Daucosterol was also found to have much higher toxicity in lung cancer cells than in noncancerous cells. As a result, daucosterol was found to be more effective for lung cancer cells because the difference between IC_50_ values in lung cancer cells (0.18 ± 0.02 mg/mL) compared to noncancerous cells (0.26 ± 0.03 mg/mL) was statistically significant.

In conclusion, this research not only contributes to the discovery of new therapeutic agents and functional foods but also fosters the conservation and sustainable use of regional flora. By creating a structured approach to holistic phytochemical profiling, it sets a new paradigm in natural product research and encourages multidisciplinary collaboration across botany, pharmacology, chemistry, and nutrition. This framework can guide future studies, leading to a deeper understanding of plant biochemistry and a more effective utilization of plant resources for human health and industry.

## 4 Materials and Methods

### 4.1 General Experimental Procedure

NMR data were recorded on a Bruker Avance, 500 MHz NMR spectrometer. HR–MS data was acquired using Thermoscientific-Thermo Q Exactive mass spectrometer. Antioxidant assays were measured using a Biotek Synergy H1 Hybrid Reader. Silica gel (60 Å, 70–230 mesh, Sigma-Aldrich), C18 (LiChroprep RP-18 (40–63 μm)), and Sephadex LH-20 were used as packing materials for open column chromatography. Chemicals for antioxidant assay purposes were acquired from Sigma-Aldrich.

### 4.2 Plant Material

**Figure 5:**
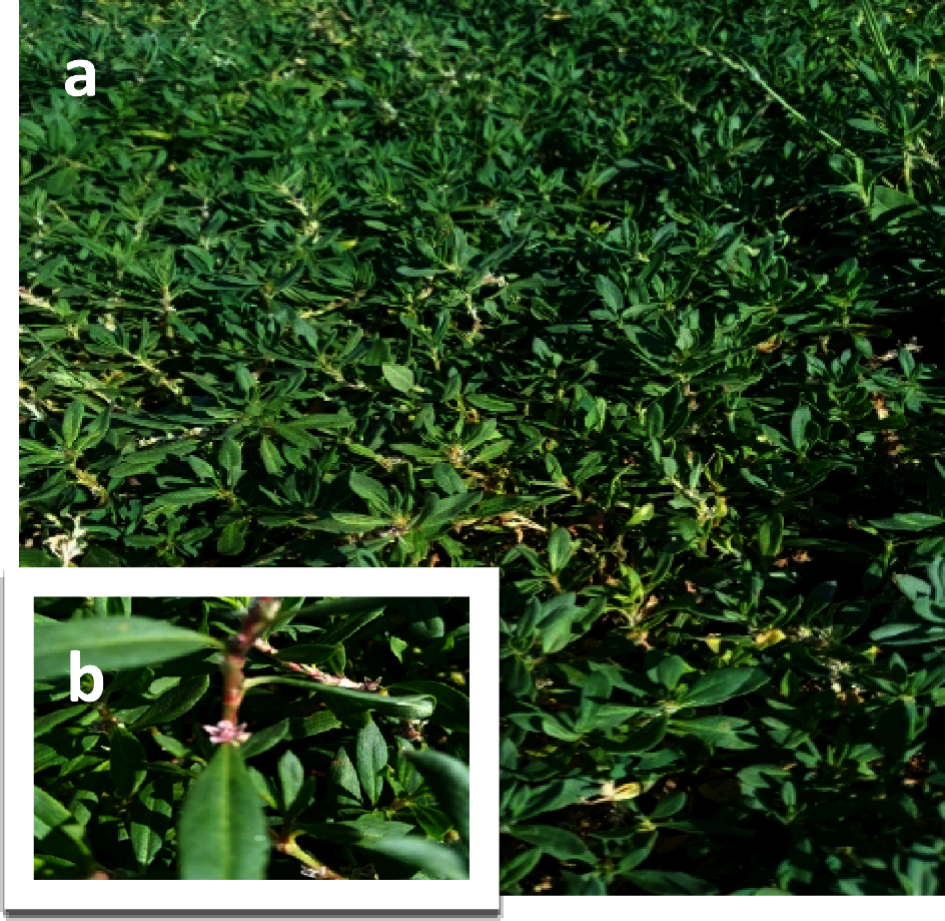
(a) The general appearance of the *P. sivasicum* and (b) Flowering branch. Photograph courtesy of Kübra Feyza Erol.

The aerial parts of *P. sivasicum* (Figure 5) were collected from Sivas, Güre at an altitude of 1700 m in June 2022 during the flowering season. The plant material was dried in the shade under controlled conditions, with a room temperature of 23 ± 2 °C, a relative humidity of 60%, and good air ventilation. The moisture content of the plant material was determined to be 7.38%. Collected specimens were identified according to the Flora of Turkey^1^ and relevant literature on the genus concerning.^27^ A voucher specimen was deposited at the Herbarium of the Department of Pharmacy, Istanbul University (ISTE no: 117503).

### 4.3 Extraction and Isolation

**Figure 6:**
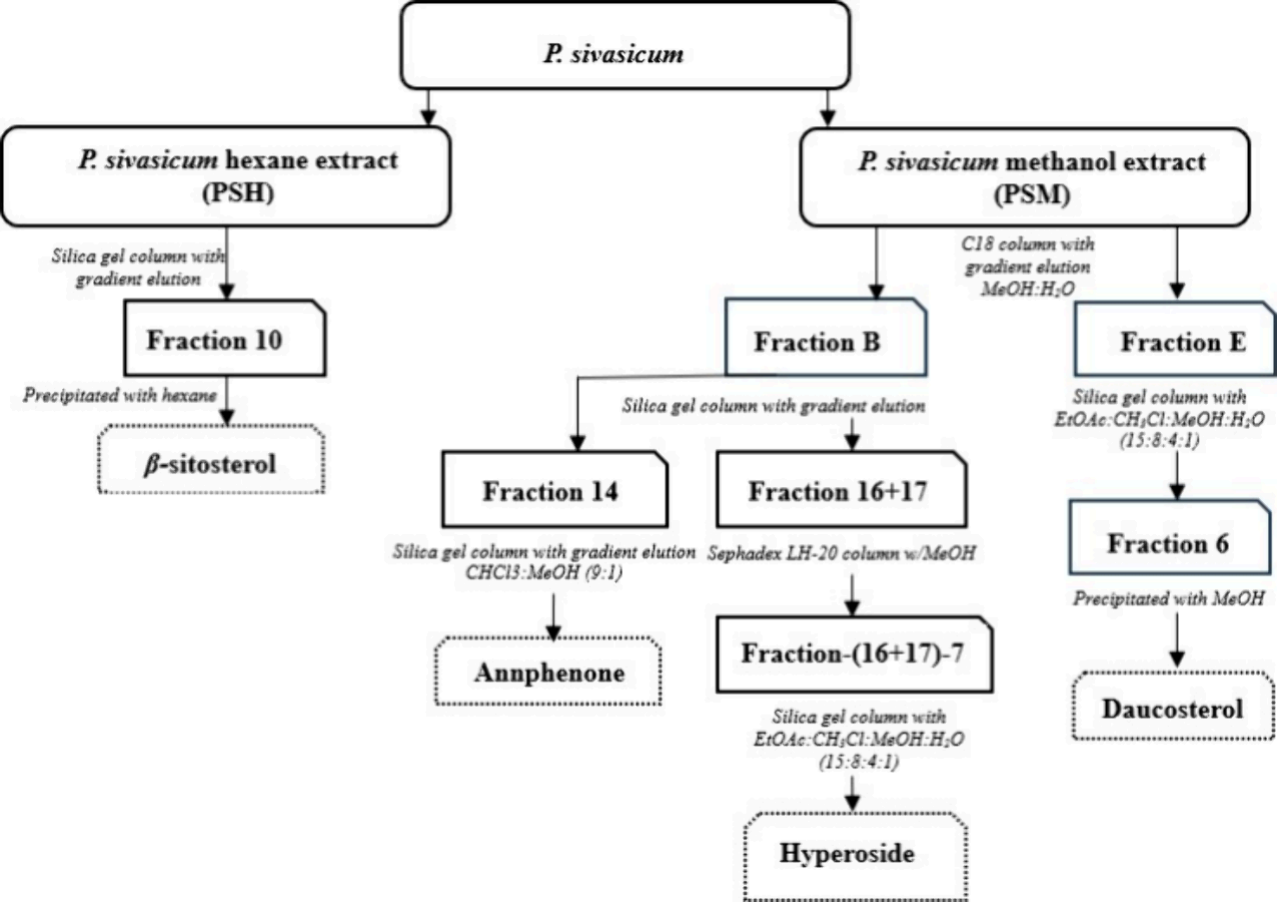
Systematic fractionation and bioactive compound isolation from *P. sivasicum*.

The dried plant sample (594 g) was pulverized and macerated at room temperature with hexane (6 × 1.2 L × 48 h), ethyl acetate (6 × 1.2 L × 48 h), chloroform (6 × 1.2 L × 48 h), and methanol (6 × 1.2 L × 48 h), successively. The obtained extracts were filtered, and solvents were evaporated under reduced pressure to yield hexane (PSH, 8.28 g), ethyl acetate (PSE, 9.74 g), chloroform (PSK, 1.90 g), and methanol extracts (PSM, 60.4 g) in the order of maceration. Additionally, the extract codes are listed in Table S1. The plant residue was dried and infused with 80 °C distilled water for 6 h, until water temperature reached 25 °C. The water was removed from the filtrate by using a freeze-dryer to obtain PSS (30.98 g) (Figure 6). Ten g of *P. sivasicum* sample was placed in a beaker, and 250 mL of distilled water was added. The plant was cooked for three hours on a hot plate similar to how natives would cook it at home. A cooked sample (PCC) was then dried by a freeze-dryer. The methanol extract (PSM, 55.0 g) was applied to column chromatography (CC) over C18 (60 × 6.5 cm), eluting under pressure with gradients of water and methanol (100:0, 75:25, 50:50, 25:75, and 0:100) to afford 5 fractions (PSMFA-PSMFE). PSMFB (20.42 g) was rechromatographed on a silica gel column (65 × 4 cm) and the elution was started with hexane: dichloromethane (100:0–0:100), proceeded with dichloromethane: methanol (100:0–0:100) in 25% increments to generate 28 subfractions. Fraction PSMFB-14 (300.1 mg) was subjected to silica gel CC (61 × 2.5 cm) eluting with a mixture of chloroform: methanol (9:1) to yield Compound **1** (55.92 mg). Fraction PSMFB-16 (332 mg) and PSMFB-17 (600 mg) were fractionated by a Sephadex LH-20 column (42 × 2 cm) with methanol, separately. Similar subfractions were combined, and the resulting subfraction PSMFB-(16–17)-7 (535.46 mg) was purified on a silica gel column (64 × 2.5 cm) using a mixture of EtOAc: CHCl_3_: MeOH: Water (15: 8: 4: 1) as eluent to yield Compound **2** (35 mg). PSMFE (3.4 g) was applied to silica gel CC (52 × 4 cm) and eluted with a mixture of EtOAc: CHCl_3_: MeOH: Water (15: 8: 4: 1) to give 23 fractions. MeOH was added to Fr. PSMFE-6 to precipitate Compound **3** (9 mg) as a white amorphous solid. The hexane extract (PSH, 7.0 g) was subjected to silica gel CC and the elution was started with 100% hexane. The composition of the mobile phase was changed gradually (100:0–0:100) to increase polarity as DCM and MeOH were introduced in 25% increments consecutively. The elution was completed with 100% MeOH to yield 13 subfractions. Compound **4** was precipitated and isolated as needle-like crystals through dissolving subfraction PSHF10 in hexane.

### 4.4 Quantification of the Phenolic Compounds by LC-HRESIMS Analysis

The Orbitrap Q-Exactive HRMS system (Thermo Fisher Scientific Inc., Waltham, MA) was integrated with a heated electrospray ionization (ESI) source. The source was operated in both positive and negative ionization modes coupled with an HPLC system for enhanced analytical performance. The gradient program conditions were performed as described previously.^65^

### 4.5 Fatty Acid Profiling by GC–MS Analysis

Two mL portion of 0.5 N NaOH was added into hexane extract of *P. sivasicum* and mixed to dissolve in a 50 °C water bath. After the addition of 2 mL of BF_3_:CH_3_OH, the mixture was let to boil at 80 °C for 2–3 min and allowed to cool. The volume was made up to 25 mL with saturated NaCl and the procedure was finished by liquid–liquid extraction with hexane.

### 4.6 In Vitro Antioxidant Activity Assays

The ABTS^·+^,^66^ DPPH^·^,^67^ metal chelating,^68^ CUPRAC,^69^ and β-carotene linoleic acid^70^ bleaching assays of the hexane (PSH), ethyl acetate (PSE), chloroform (PSK) and methanol (PSM) extracts as well as water infusion (PSS) and cooked (PSC) plant samples were tested according to the methods previously described.^71−73^

### 4.7 In Vitro Cytotoxic Activity

#### 4.7.1 Cell Culture and Treatment

The A549 (human lung carcinoma epithelial cells, ATCC, CCL-185), MCF-7 (human breast cancer cells, ATCC, HTB-22), and CCD-1079Sk (human normal skin fibroblast epithelial cells, ATCC, CRL-2097) cell lines were initially seeded into culture flasks and grown in DMEM supplemented with 10% FBS (Sigma-Aldrich, St Louis, MO), 1% penicillin/streptomycin (Thermo Fisher Scientific, Carlsbad, CA). The cell lines were maintained in a humidified atmosphere of 5% CO_2_ at a temperature of 37 °C. The culture medium was changed every two days until the cells reached 80% confluence.

#### 4.7.2 Cell Viability Assay

3-(4,5-dimethyl-2-thiazolyl)-2,5-diphenyl-2H-tetrazolium bromide (MTT) was used to assess the cytotoxicity of hyperoside, annphenone, daucosterol, and *P. sivasicum* methanol extract. Cells were grown in 96-well plates at a density of 5 × 10^3^ cells per well. In total, 70 to 80% of confluent cells (after 24 h) were treated with different concentrations of compounds (prepared in a medium containing 1% DMSO) in the growth medium for 24 h. At the end of the incubation periods, MTT solution was added to the wells to give a final concentration of 0.5 mg/mL. The cells were then incubated at 37 °C for a further 3.5 h. To solubilize the formazan crystals, 100 μL of DMSO was added to each well. The optical density was read at 570 nm by using an ELISA microplate reader and directly correlated with the number of viable cells. For each concentration, the data for which the measurement was repeated at least three times were compared. The relative % cell viability and the IC_50_ value were determined by plotting the graph as a function of concentration. Untreated cells were considered as control, and medium without cells was considered as background control. To determine the cytotoxic effect of compounds on cell viability, cells that were not treated with compounds were considered 100% viable, and cell viability was calculated according to the following formula: % Cell Viability = Sample/Control × 100.

### 4.8 Statistical Analyses

All data on bioactivity tests were averages of triplicate analyses. Activity assays were carried out at six concentrations, and the results are given as the IC_50_ values. The data were analyzed using One-way ANOVA followed by Tukey’s post hoc test. *p* < 0.05, *p* < 0.001 and *p* < 0.0001 were considered statistically significant. Data were recorded as mean ± SEM (standard error of the mean). Significant differences between means were determined by the one-way ANOVA, and *p* values < 0.05 were regarded as significant. IBM SPSS statistical software (Version: 28.0.0.0 (190)) and GraphPad Prism 10.2 were used for statistical analysis.

## Supporting Information Available

The list of standards used for LC-HRESIMS analysis; validation and uncertainty parameters for phenolic compounds; 1D-, 2D-NMR and HRESIMS spectra of isolates; graphs for antioxidant activity test results; a graph for IC_50_ values (PDF)

## Notes

This article does not contain any tests conducted on animal and human subjects.
