# Supplementary material for: Isolation and Characterization of Secondary Metabolites from Endemic and Edible Polygonum sivasicum with In Vitro Antioxidant and Cytotoxic Activities
Source: ACS Omega. 2025 Feb 26;10(9):9756–67. doi: 10.1021/acsomega.5c00438 (PMC11904701; doi:10.1021/acsomega.5c00438)
Supplement: Supplementary file 1 — ao5c00438_si_001.pdf [file ao5c00438_si_001.pdf]

Supplementary material

Isolation and Characterization of Secondary Metabolites from Endemic & Edible *Polygonum sivasicum* with *in vitro* Antioxidant & Cytotoxic Activities

Humeyra Karakas[1] • Zeynep Cagman[2] • Cagla Kizilarslan-Hancer[3] • Ebru Erol[4]*

[1]Department of Pharmacognosy and Natural Products Chemistry, Health Sciences Institute, Bezmialem Vakif University, 34093, Istanbul, Türkiye; [2]Department of Biochemistry, Faculty of Pharmacy, Bezmialem Vakif University, 34093, Istanbul, Türkiye; [3]Department of Pharmaceutical Botany, Faculty of Pharmacy, Bezmialem Vakif University, 34093, Istanbul, Türkiye; [4]Department of Analytical Chemistry, Faculty of Pharmacy, Bezmialem Vakif University, 34093, Istanbul, Türkiye.

*ebruerol@bezmialem.edu.tr

Isolation and Characterization of Secondary Metabolites from Endemic & Edible *Polygonum sivasicum* with *in vitro* Antioxidant & Cytotoxic Activities

Abstract

Polygonum sivasicum Kit Tan & Yildiz, one of the eight endemic *Polygonum* species in Türkiye, belongs to the Polygonaceae family. Preliminary phytochemical investigation of methanol and hexane extracts of *P. sivasicum* resulted in four compounds, namely annphenone (**1**), hyperoside (**2**), daucosterol (**3**), and *β*-sitosterol (**4**). Their structures were elucidated by 1D-, 2D-NMR and HRESIMS analyses. This study signifies the first isolation of annphenone from *Polygonum* genus. Antioxidant capabilities of different extracts of *P. sivasicum* were carried out using $DPPH^{\cdot}$, $ABTS^{\cdot+}$, CUPRAC, metal chelating and *β*-carotene linoleic acid bleaching assays, and their effectiveness was quantified through IC_{50} values. Furthermore, 27 phenolic compounds were identified using LC-HRESIMS from methanol extract, which has the highest antioxidant activity among *P. sivasicum* extracts. The major phenolic constituents identified were hyperoside (4535.0 µg/g extract), rutin (4387.4 µg/g extract), and chlorogenic acid (3306.6 µg/g extract). GC-MS analysis determined palmitic acid, *α*-linolenic acid and 8, 11-octadecadieonic acid as major fatty acids in hexane extract. The cell viability profile of *P. sivasicum* methanol extract and its isolates hyperoside, annphenone, and daucosterol was evaluated on fibroblast (CCD-1079Sk), breast carcinoma (MCF-7) and lung carcinoma (A549) cell lines. Annphenone exhibited IC_{50} values of 0.25±0.01 mg/mL against the A549 cell line, and 0.36±0.02 mg/mL against the MCF-7 cell line. The selective cytotoxicity observed for daucosterol against the A549 cell line, with a high selectivity index of 1.44, underscores its potential as a promising candidate for drug development. The study establishes a framework integrating phytochemical profiling with biological assays to identify therapeutic agents from endemic plants.

Keywords: *Polygonum sivasicum* • LC-HRESIMS • antioxidant activity • annphenone • NMR • cytotoxicity

List of supplementary material

Table S1: List of abbreviations. 5

Table S2: The list of standards used for LC-HRESIMS based phenolic profiling. 6

Table S3: The list of standards used for LC-HRESIMS based phenolic profiling. 7

Figure S1: ^{1}H- NMR (500 MHz, CD_3OD) spectrum of compound (**1**). 8

Figure S2: ^{13}C- NMR (125 MHz, CD_3OD) spectrum of compound (**1**). 9

Figure S3: HSQC spectrum of compound (**1**)……………………………………...…..10

Figure S4: HMBC spectrum of compound (**1**)………………...…………………...…..11

Figure S5: COSY spectrum of compound (**1**). 12

Figure S6: HRESIMS **s**pectrum of compound (**1**). 13

Figure S7: ^{1}H- NMR (500 MHz, DMSO-d_6) spectrum of compound (**2**). 14

Figure S8: ^{13}C- NMR (125 MHz, DMSO-d_6) spectrum of compound (**2**). 15

Figure S9: HSQC spectrum of compound (**2**). 16

Figure S10: HMBC spectrum of compound (**2**). 17

Figure S11: ^{1}H- NMR (500 MHz, DMSO-d_6) spectrum of compound (**3**). 18

Figure S12: ^{13}C- NMR (125 MHz, DMSO-d_6) spectrum of compound (**3**). 19

Figure S13: HSQC spectrum of compound (**3**). 20

Figure S14: HMBC spectrum of compound (**3**). 21

Figure S15: COSY spectrum of compound (**3**). 22

Figure S16: ^{1}H- NMR (500 MHz, $CDCl_3$) spectrum of compound (**4**). 23

Figure S17: ^{1}H- NMR spectrum of compound (**4**) (top) in comparison to *β*-sitosterol isolated from *Caulerpa cylindracea* (bottom). 24

Figure S18: ^{1}H- NMR spectrum of compound (**4**) (top) in comparison to *β*-sitosterol isolated from *C. cylindracea* (bottom)-close-up view..25

Figure S19: ^{13}C- NMR (125 MHz, $CDCl_3$) spectrum of compound (**4**). 26

Figure S20. Antioxidant activity results of polar extracts in four different assays. 27

Figure S21. Antioxidant activity results of nonpolar samples in four different assays...28

Figure S22. Metal chelating assay results of six different extracts and the standard.....29

Figure S23. IC_{50} values of compounds on CCD-1079Sk, MCF-7 and A549 cells lines after 24 h of treatment...30

Table S1: List of abbreviations

ABTS	2,2'-azino-bis(3-ethylbenzothiazoline-6-sulfonic acid)
CUPRAC	Cupric Reducing Antioxidant Capacity
DPPH	1,1-Diphenyl-2-picrylhydrazyl
IC_{50}	Half-maximal inhibitory concentration
PSC	*P. sivasicum* cooked sample
PSE	*P. sivasicum* ethyl acetate extract
PSH	*P. sivasicum* hexane extract
PSM	*P. sivasicum* methanol extract
PSS	*P. sivasicum* water extract

Table S2: The list of standards used for LC-HRESIMS based phenolic profiling.

Ascorbic acid	Hesperidin	Kaempferol
(-)-Epigallocatechin	Rutin	3'-*O*-methyl quercetin
(-)-Epigallocatechin gallate	Syringic acid	Apigenin
(+)-Catechin	Rosmarinic acid	Hispidulin
Chlorogenic acid	Hyperoside	Isosakuranetin
Fumaric acid	Dihydrokaempferol	Penduletin
(-)-Epicatechin	Oleuropein	Glycyrrhizic acid
(-)-Epicatechin gallate	Apigenin 7-glucoside	Sinensetin
Verbascoside	Ellagic acid	Caffeic acid phenethyl ester
Chicoric acid	Quercitrin	Rhamnocitrin
Orientin	Myricetin	Chrysin
Caffeic acid	Nepetin-7-glucoside	Acacetin
Caffeine	Scutellarein	Quillaic acid
(+)-t*rans* Taxifolin	Quercetin	Sarsasapogenin
Luteolin-7-rutinoside	Herniarin	Gypsogenic acid
Vanillic acid	Salicylic acid	Emodin
Naringin	Naringenin	Shatavarin
Sinapinic acid	Luteolin	Hederagenin
Luteolin 7-glucoside	Nepetin	(-)- Caryophyllene oxide
p-Coumaric acid	Genistein	Kaempferol

Table S3: Validation and uncertainty parameters for phenolic compounds.

Compound	U (%)	Molecular Formula	*m/z*	Ionization mode	Linear range	Linear regression equation	LOD/LOQ	R²	Recovery	%RSD
Ascorbic acid	3,94	$C_6H_8O_6$	175,0248	Negative	0.5-10	y=0.00347x-0.00137	0.39/1.29	0,9988	96,2	2,93
(-)-Epigallocatechin gallate	3,76	$C_{22}H_{18}O_{11}$	459,0922	Positive	0.3-7	y=0.00182x+0.000026	0.1/0.33	0,9989	94,76	4,20
Chlorogenic acid	3,58	$C_{16}H_{18}O_9$	353,0878	Negative	0.05-10	y=0.00817x+0.000163	0.02/0.06	0,9994	96,68	3,93
Pyrogallol	4,50	$C_6H_6O_3$	125,02442	Negative	0.5-10	y=0.5283x-0.06866	0,9954	0.35/1.17	98,38	5,51
(-)-Epicatechin gallate	3,05	$C_{22}H_{18}O_{10}$	441,0827	Negative	0.05-10	y=0.00788x-0.0001875	0.01/0.03	0,9995	96,54	3,20
Orientin	3,67	$C_{21}H_{20}O_{11}$	447,0933	Negative	0.1-10	y=0.00757x+0.000347	0.01/0.03	0,9993	96,22	4,16
Caffeic acid	3,74	$C_9H_8O_4$	179,0350	Negative	0.3-10	y=0.0304x+0.00366	0.08/0.27	0,9993	94,51	3,23
3,4-Dihydroxybenzaldehyde	3,79	$C_7H_6O_3$	137,02442	Negative	0.5-10	y=1.343x+0.6441	0,9962	0.53/1.77	98,67	4,64
(+)-*trans* taxifolin	3,35	$C_{15}H_{12}O_7$	303,0510	Negative	0.3-10	y=0.0289x+0.00537	0.01/0.03	0,9978	91,66	3,26
Vanilic acid	3,49	$C_8H_8O_4$	167,0350	Negative	0.3-10	y=0.00133x+0.0003456	0.1/0.33	0,9997	98,66	4,74
Luteolin-7-glycoside	3,63	$C_{21}H_{20}O_{11}$	447,09328	Negative	0.5-10	y=0.02067x-0.002533	0,9984	0.17/0.58	100,46	4,45
Rutin	3,07	$C_{27}H_{30}O_{16}$	609,1461	Negative	0.05-10	y=0.00329x-0.00005576	0.01/0.03	0,999	96,97	4,12
Hyperoside	3,46	$C_{21}H_{20}O_{12}$	463,0882	Negative	0.05-10	y=0.0072x-0.00003096	0.01/0.03	0,9995	96,62	3,77
Apigenin 7-glucoside	3,59	$C_{21}H_{20}O_{10}$	431,0984	Negative	0.3-7	y=0.0246x+0.00306	0.01/0.03	0,9962	96,07	4,61
Ellagic acid	4,20	$C_{14}H_6O_8$	300,9990	Negative	0.05-10	y=0.0085x-0.000612	0.03/1	0,9994	101,49	3,90
Quercitrin	3,78	$C_{21}H_{20}O_{11}$	447,0933	Negative	0.05-10	y=0.0179+0.0003331	0.01/0.03	0,999	97	4,76
Myricetin	4,18	$C_{15}H_{10}O_8$	317,0303	Negative	0.1-10	y=0.0202x+0.00165	0.01/0.03	0,9993	100,1	4,17
Quercetin	2,95	$C_{15}H_{10}O_7$	301,0354	Negative	0.1-10	y=0.0509x+0.00467	0.01/0.03	0,9978	96,41	2,90
Salicylic acid	1,89	$C_7H_6O_3$	137,0244	Negative	0.3-10	y=0.0361x+0.00245	0.01/0.03	0,9982	92,88	3,97
Naringenin	4,20	$C_{15}H_{12}O_5$	271,0612	Negative	0.1-10	y=0.0281x+0.00182	0.01/0.03	0,9995	86,65	1,52
Luteolin	3,42	$C_{15}H_{10}O_6$	285,0405	Negative	0.1-10	y=0.117x+0.00848	0.01/0.03	0,9981	96,98	4,77
Nepetin	2,19	$C_{16}H_{12}O7$	315,0510	Negative	0.05-10	y=0.0853x+0.00269	0.01/0.03	0,9992	97,76	3,70
Chrysoeriol	2,08	$C_{16}H_{12}O_6$	299,05611	Negative	0.5-10	y=0.1023x-0.002224	0,9974	0.15/0.5	96,42	2,55
Apigenin	2,87	$C_{15}H_{10}O_5$	269,0456	Negative	0.3-10	y=0.104x+0.0199	0.01/0.03	0,9998	81,55	4,07
Hispidulin	3,41	$C_{16}H_{12}O_6$	301,0707	Positive	0.05-10	y=0.02614x+0.0003114	0.01/0.03	0,9993	98,36	2,91
Chrysin	3,24	$C_{15}H_{10}O_4$	253,0506	Negative	0.05-7	y=0.0964x-0.0002622	0.01/0.03	0,999	87,92	2,36
Acacetin	3,98	$C_{16}H_{12}O_5$	283,0612	Negative	0.05-7	y=0.046x+0.0001875	0.01/0.03	0,9995	87,52	3,55

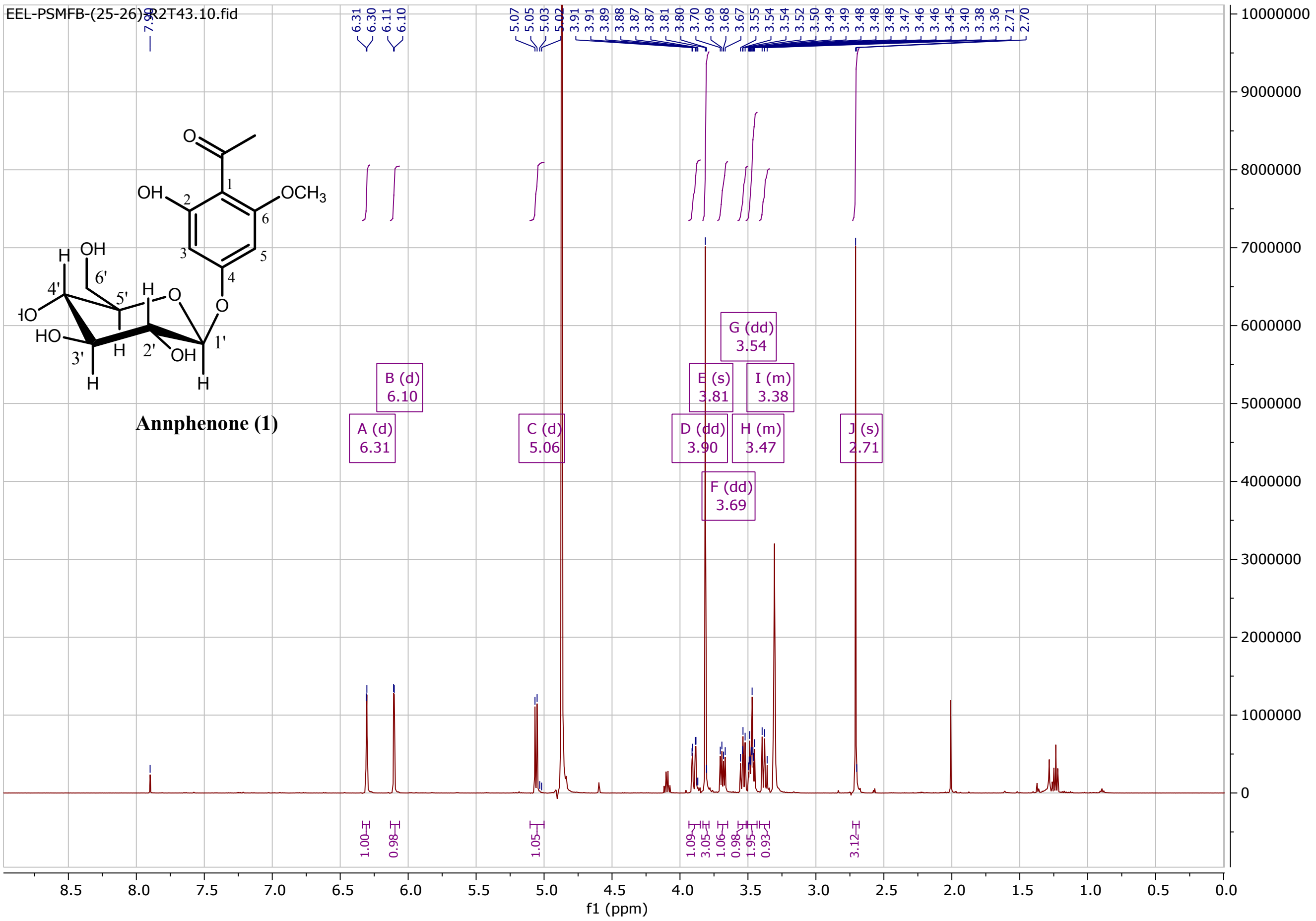

Figure S1: ^{1}H- NMR (500 MHz, CD_3OD) spectrum of compound (**1**).

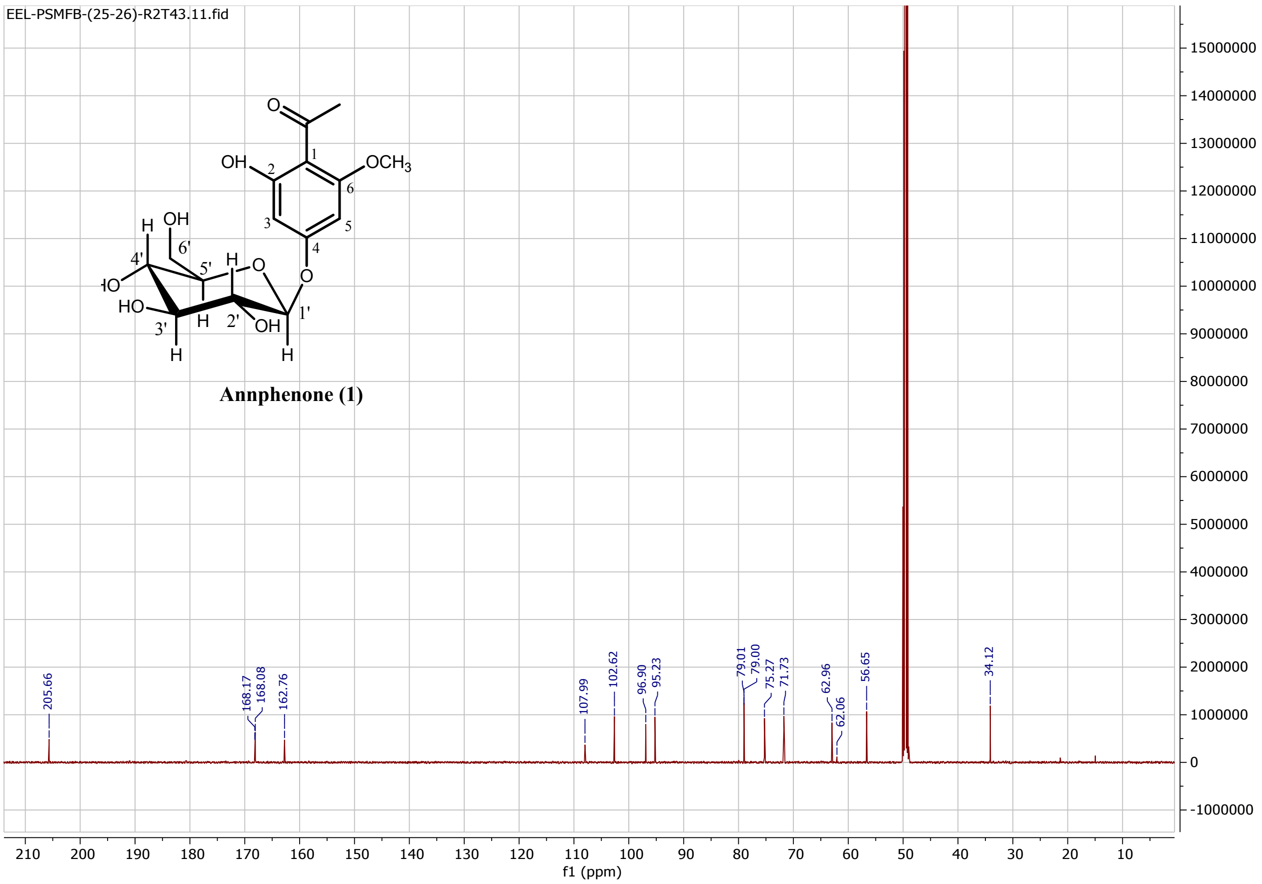

Figure S2: ^{13}C- NMR (125 MHz, CD_3OD) spectrum of compound **(1)**.

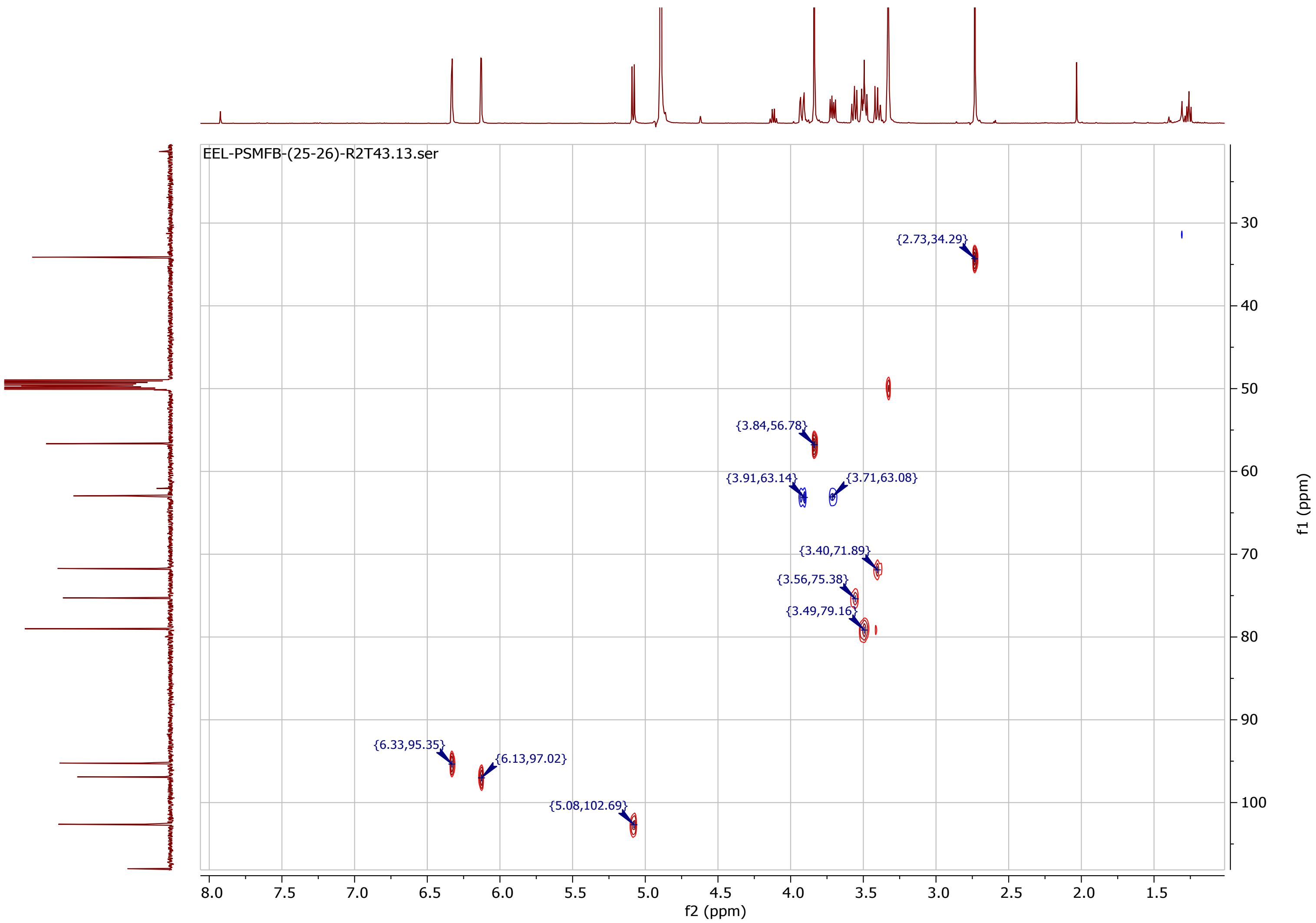

Figure S3: HSQC spectrum of compound (**1**).

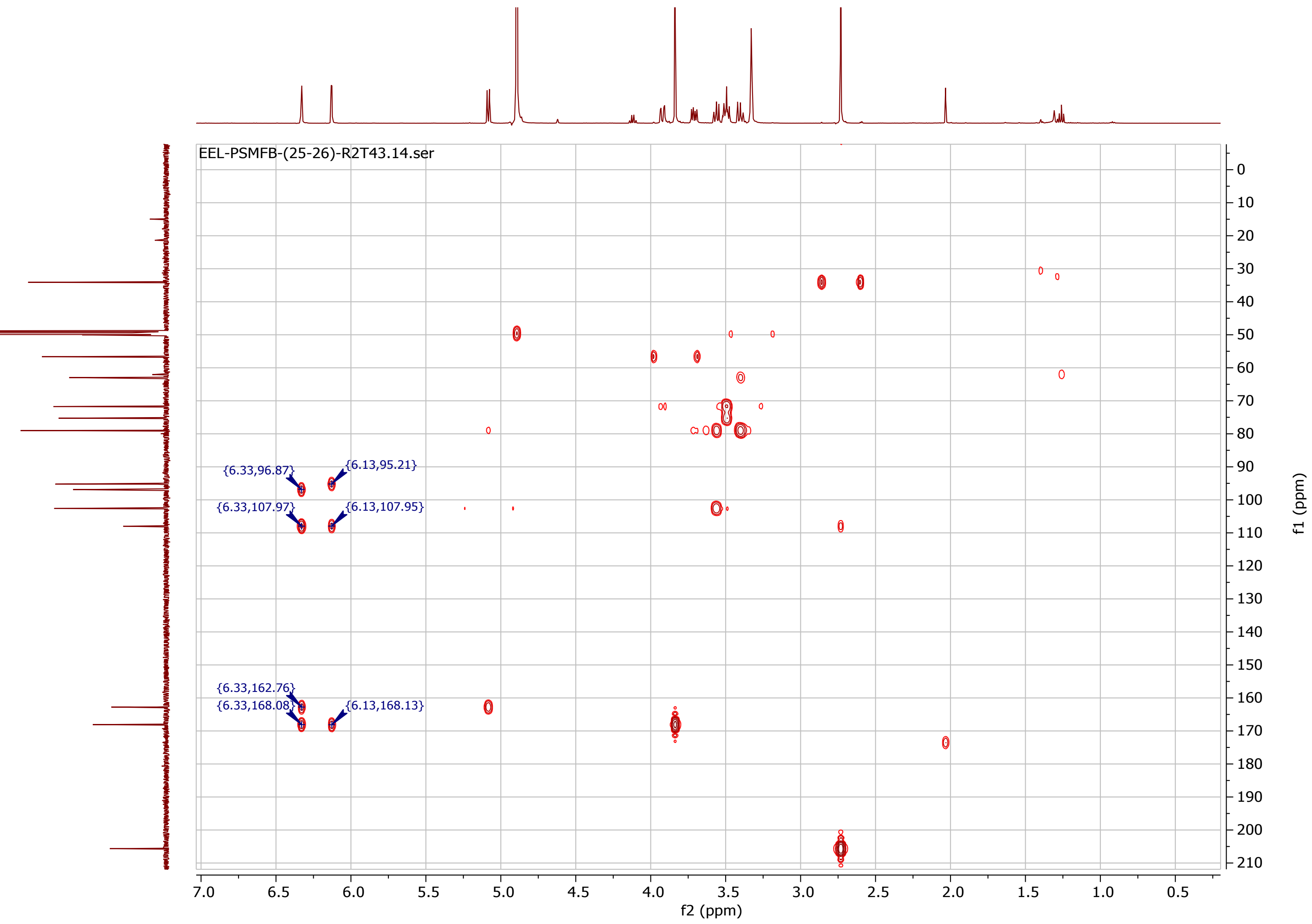

Figure S4: HMBC spectrum of compound (**1**).

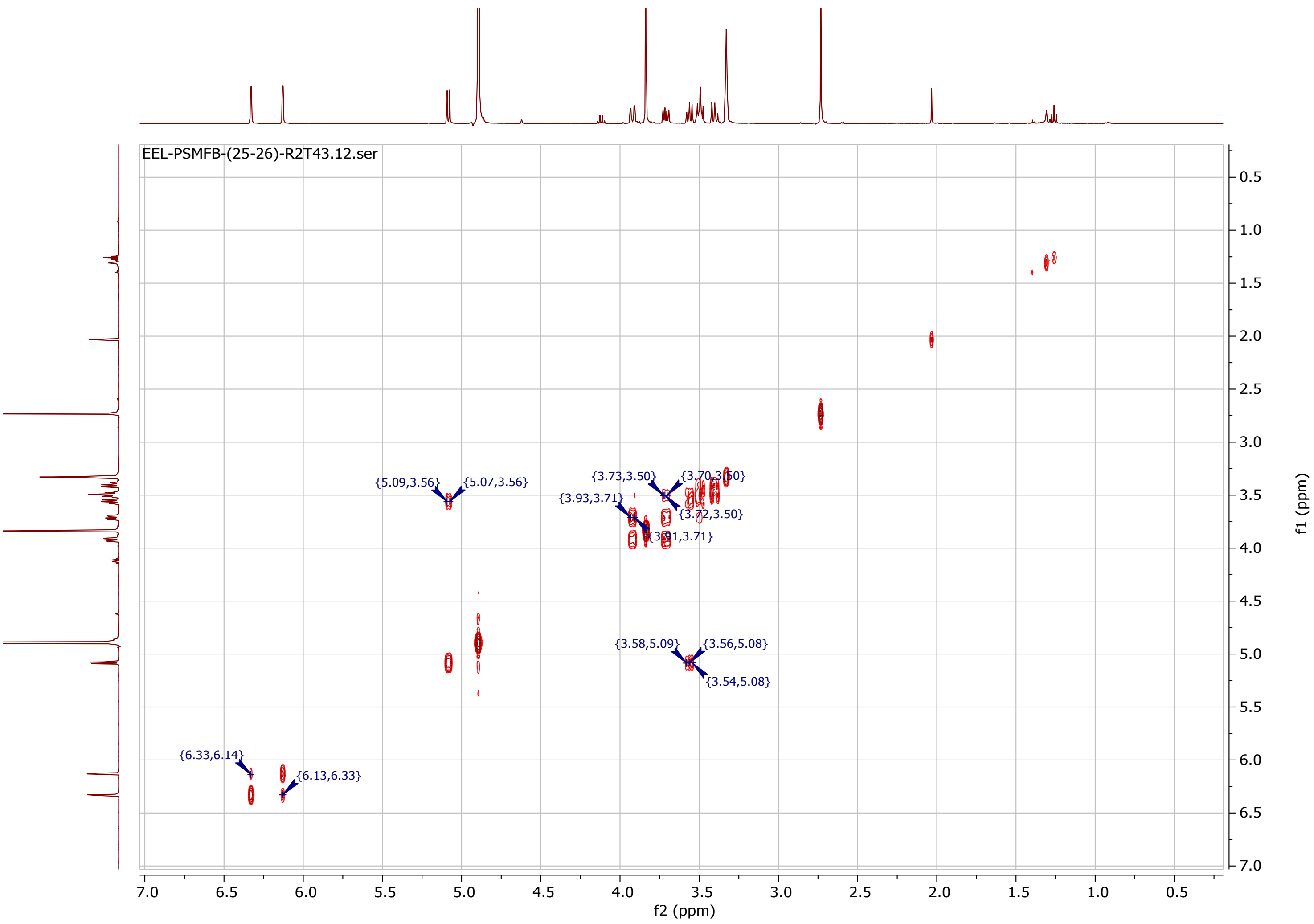

Figure S5: COSY spectrum of compound (**1**).

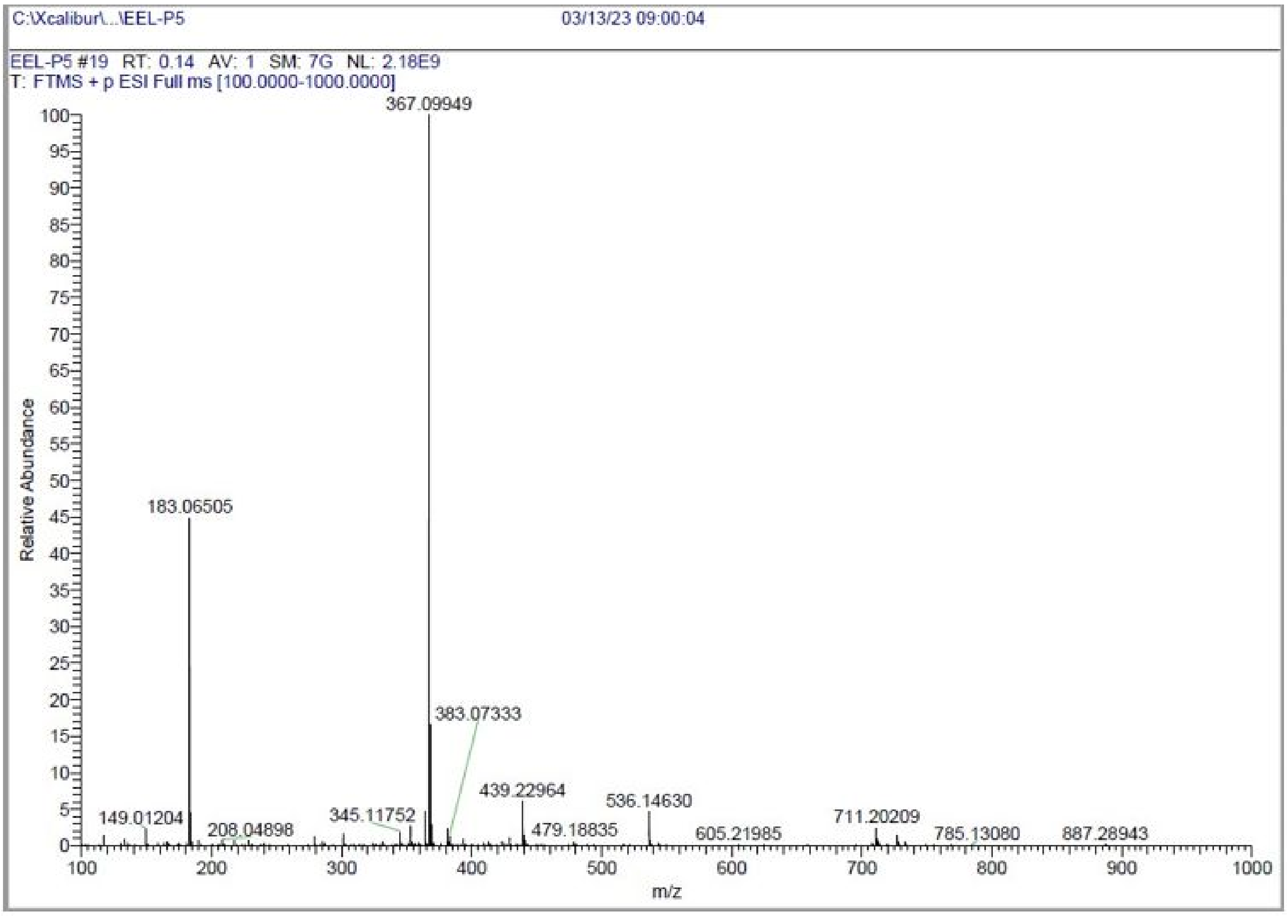

Figure S6: HRESIMS **s**pectrum of compound **(1)**.

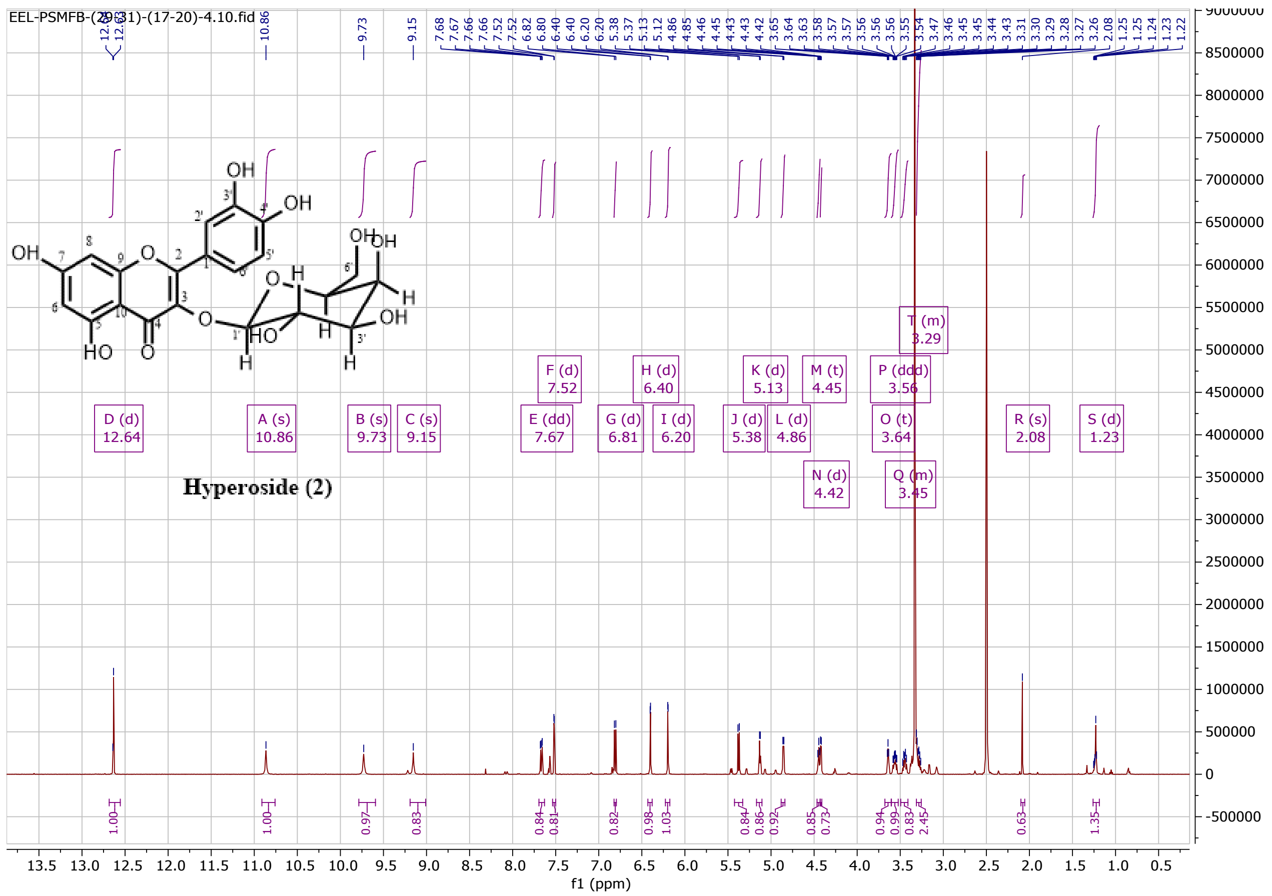

Figure S7: ^{1}H- NMR (500 MHz, DMSO-d_6) spectrum of compound (**2**).

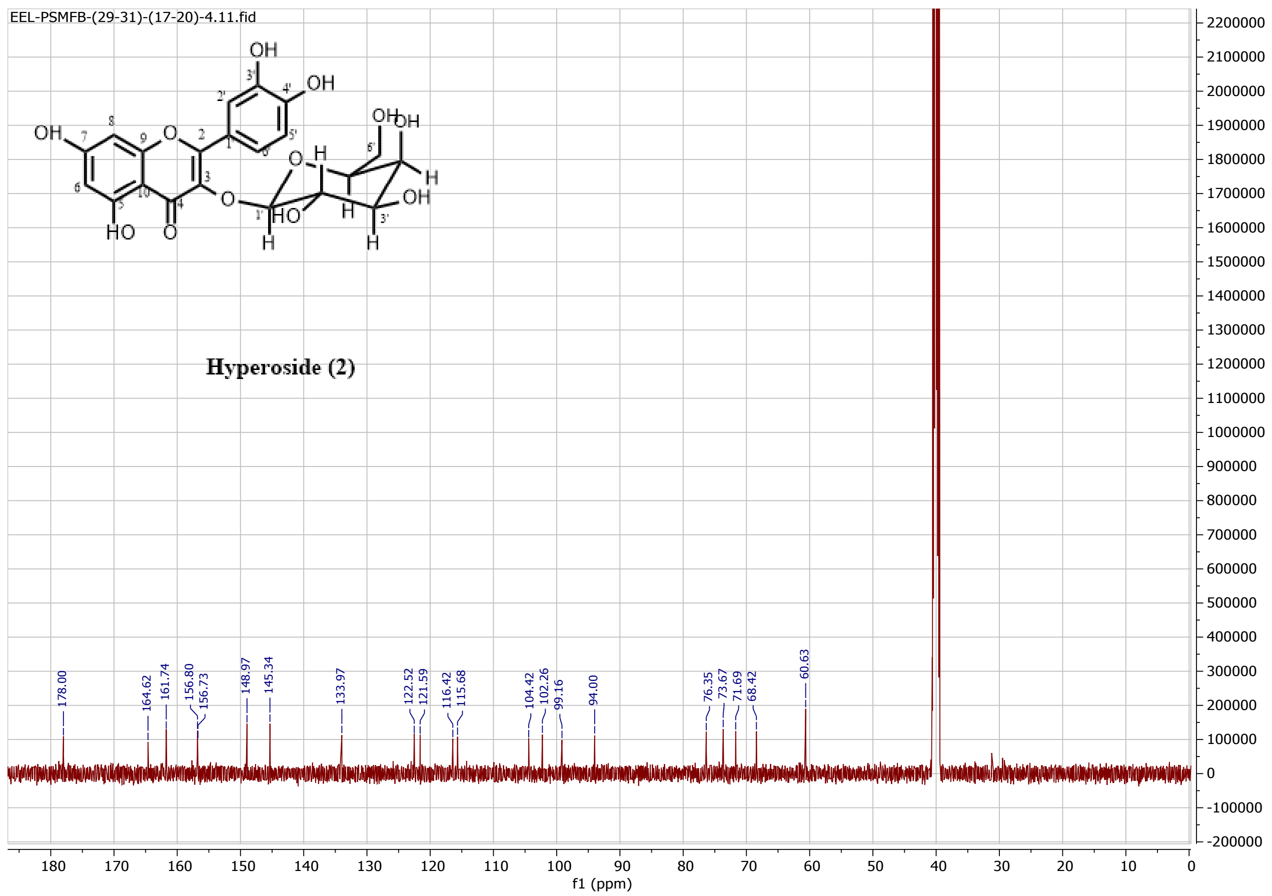

Figure S8: ^{13}C- NMR (125 MHz, DMSO-d_6) spectrum of compound (**2**).

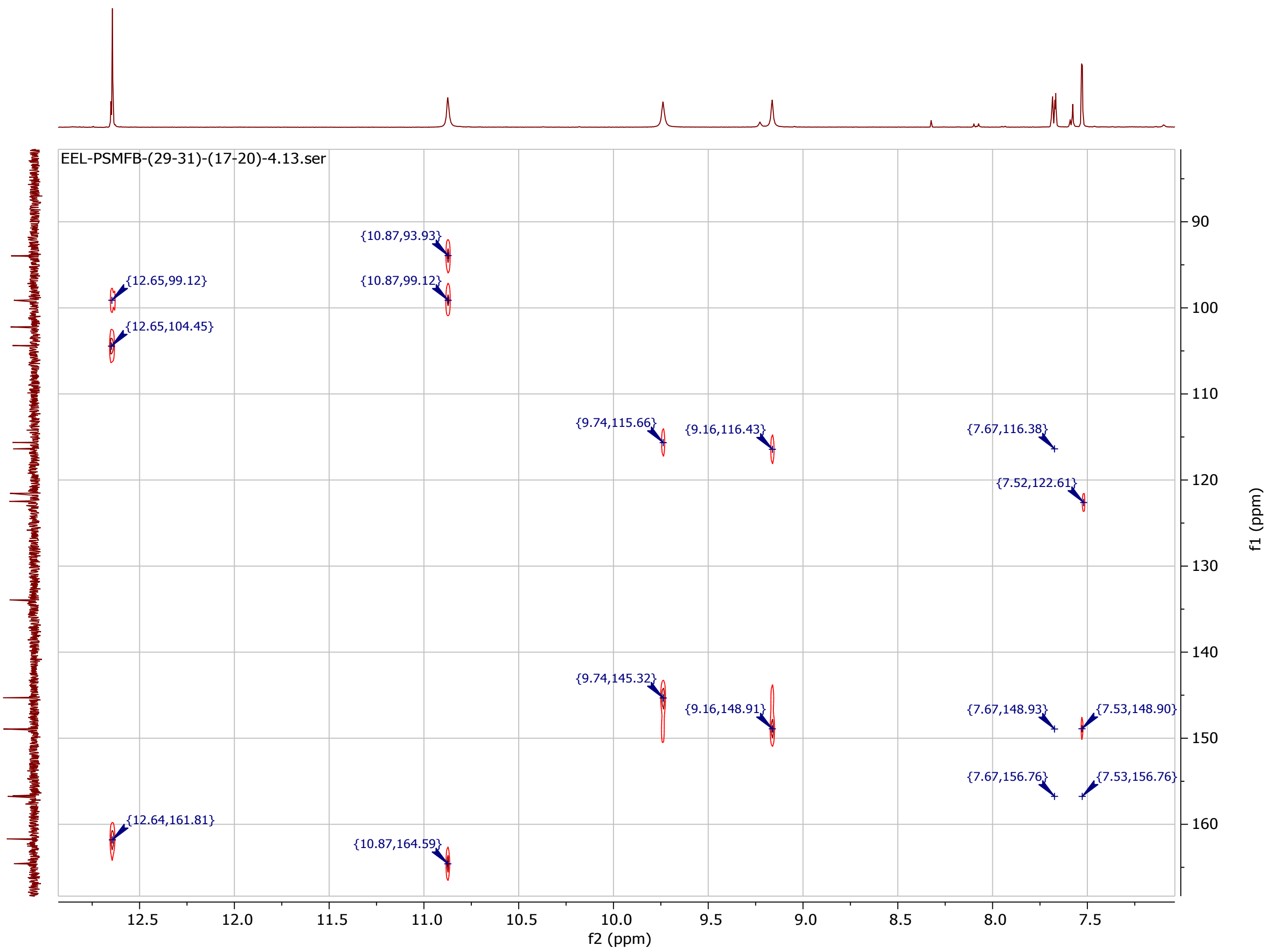

Figure S9: HSQC spectrum of compound (**2**).

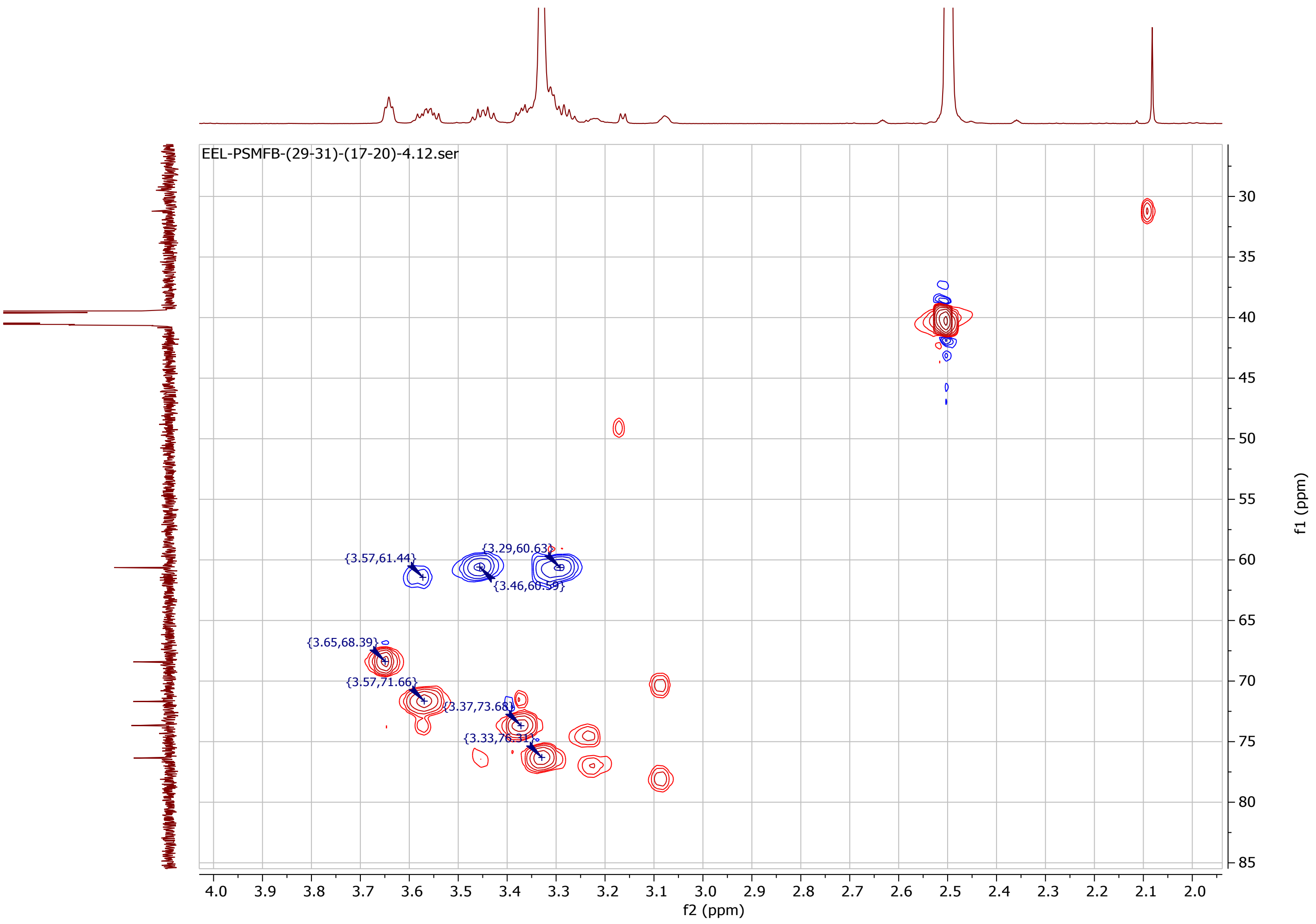

Figure S10: HMBC spectrum of compound (**2**).

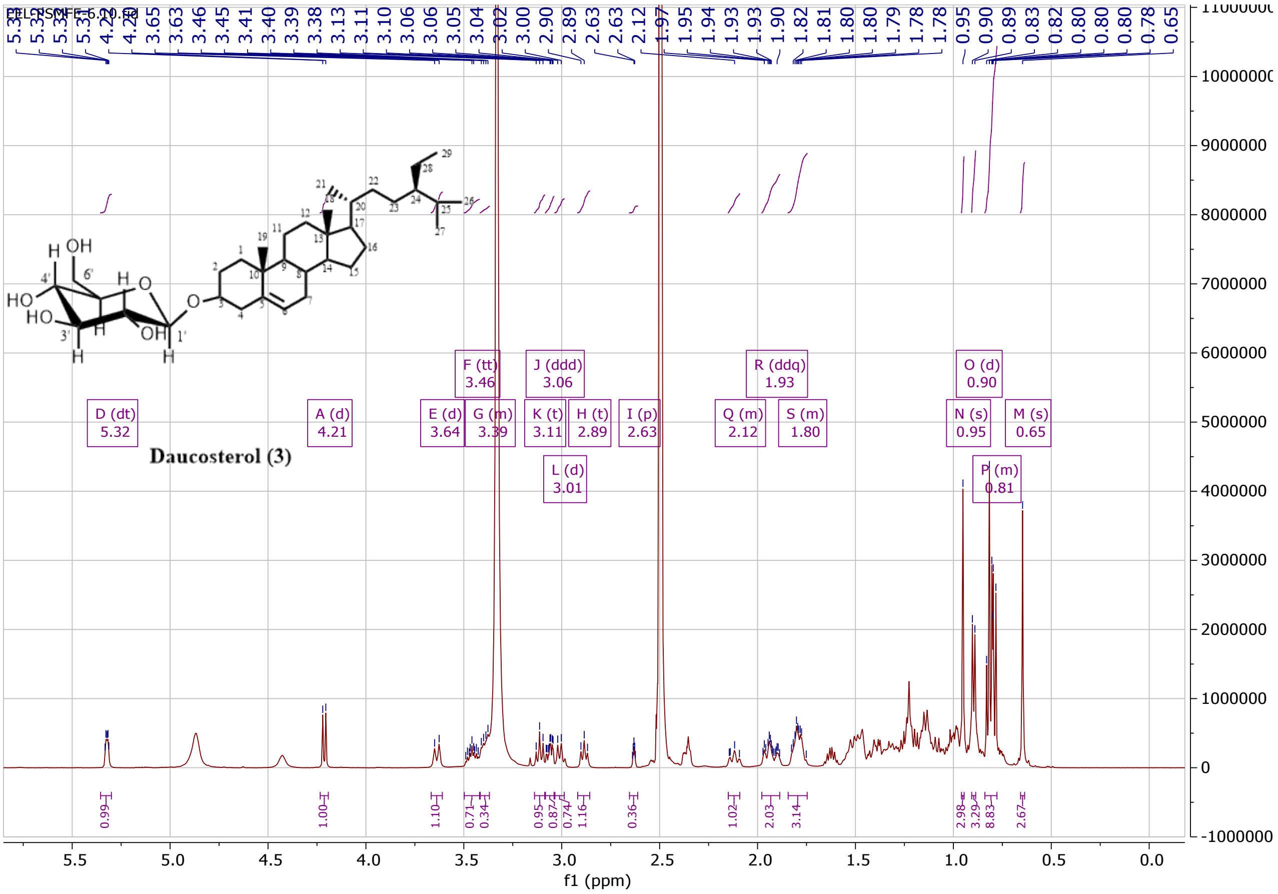

Figure S11: ^{1}H- NMR (500 MHz, DMSO-d_6) spectrum of compound (**3**).

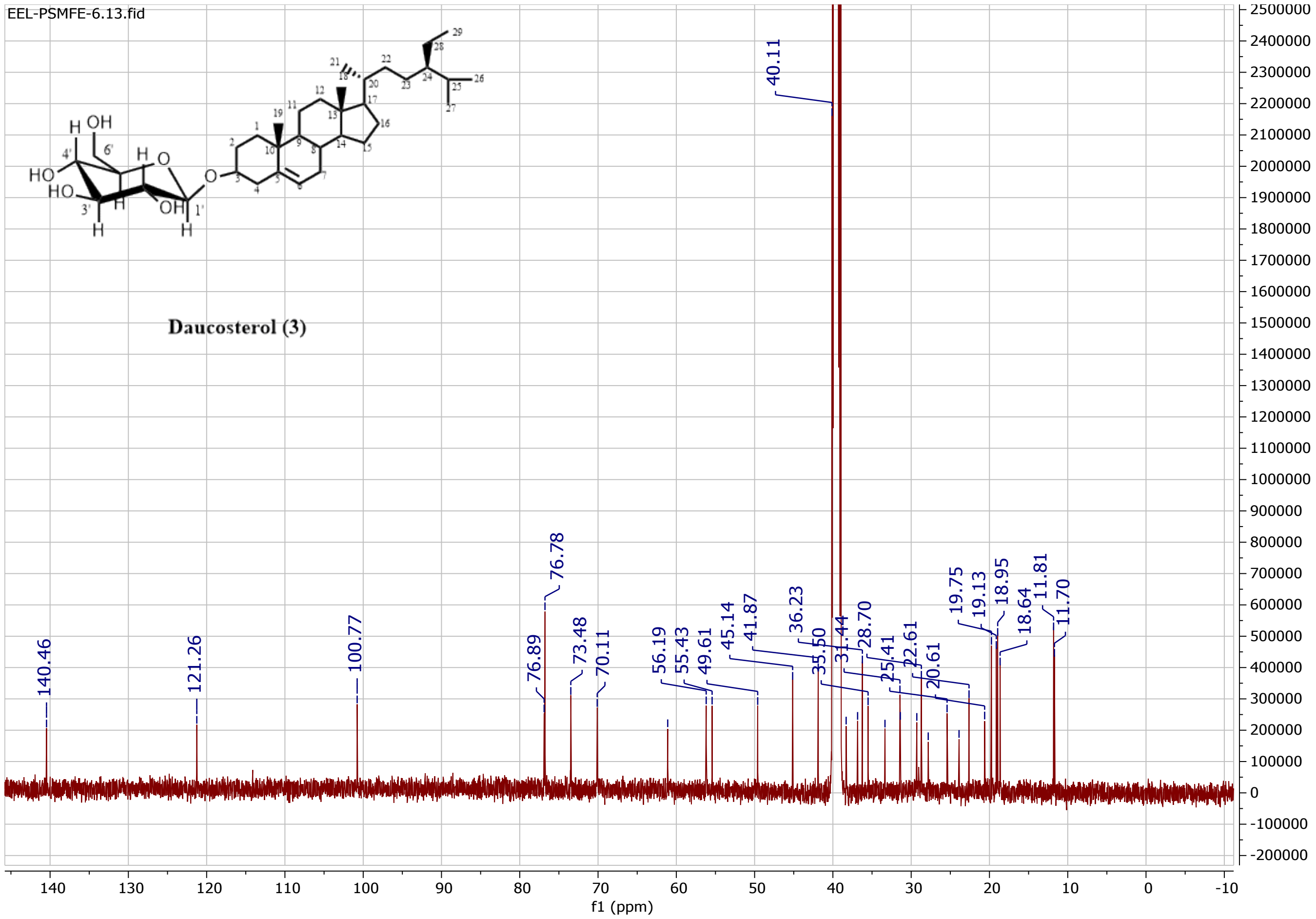

Figure S12: ^{13}C- NMR (125 MHz, DMSO-d6) spectrum of compound (**3**).

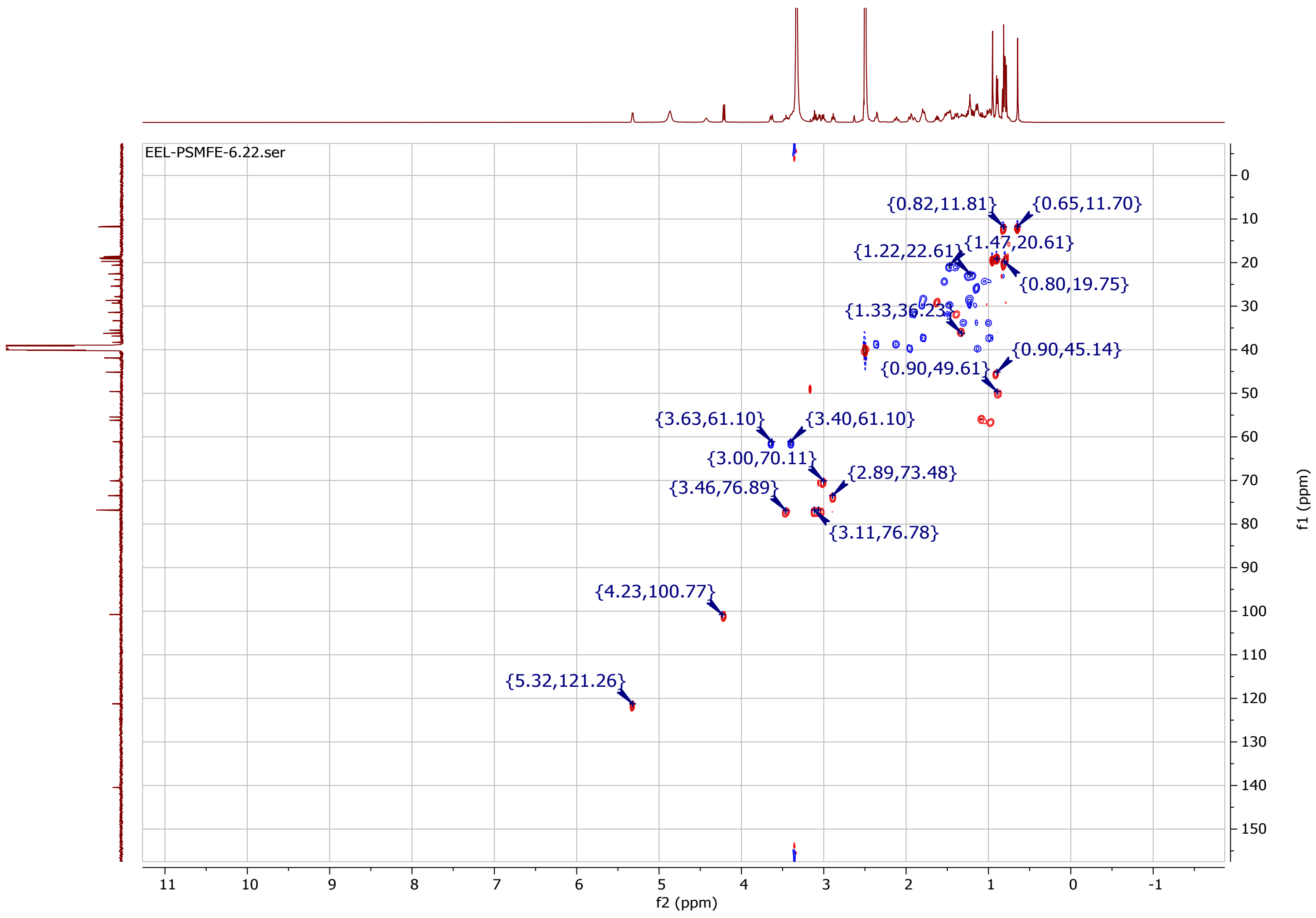

Figure S13: HSQC spectrum of compound (**3**).

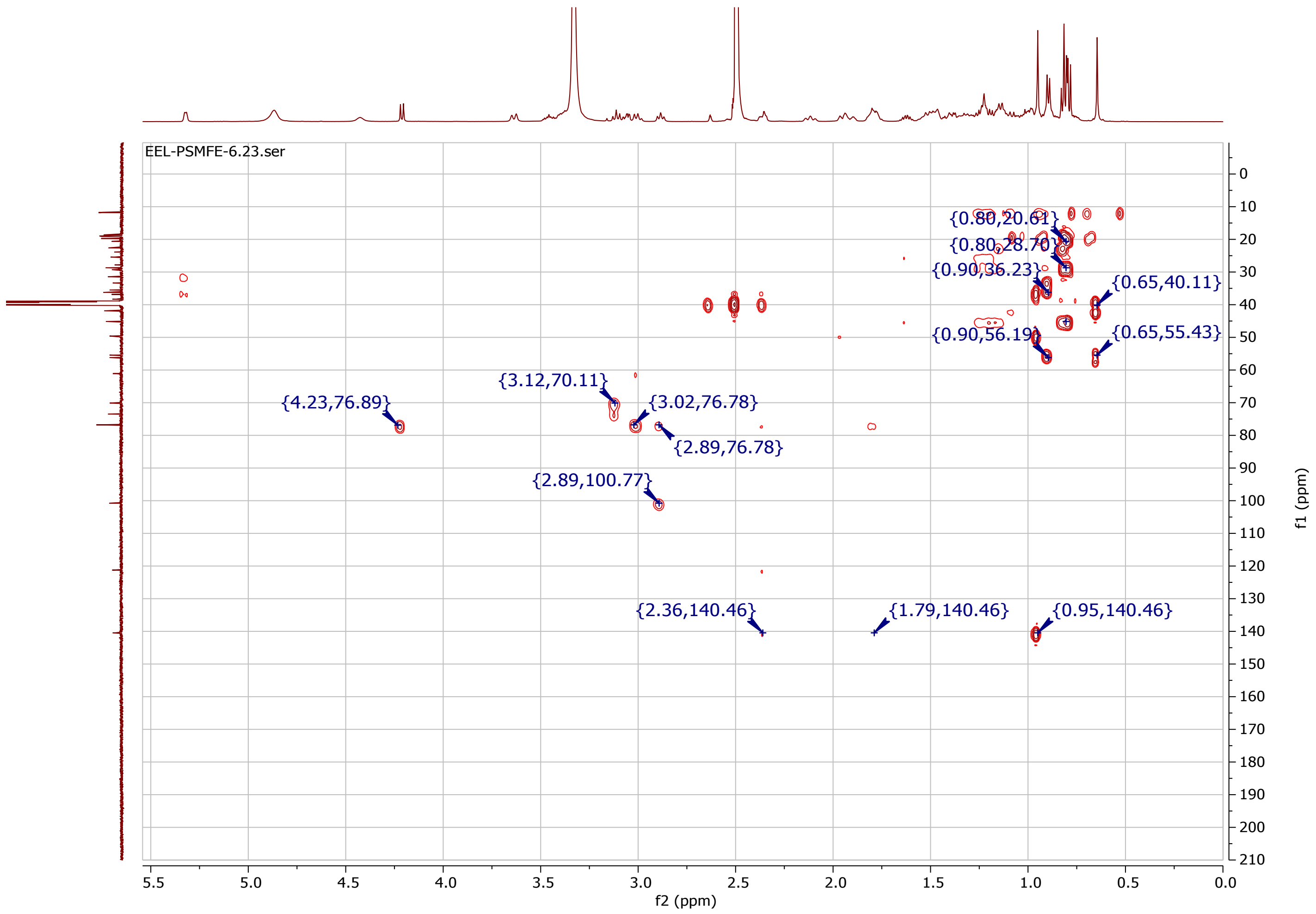

Figure S14: HMBC spectrum of compound (**3**).

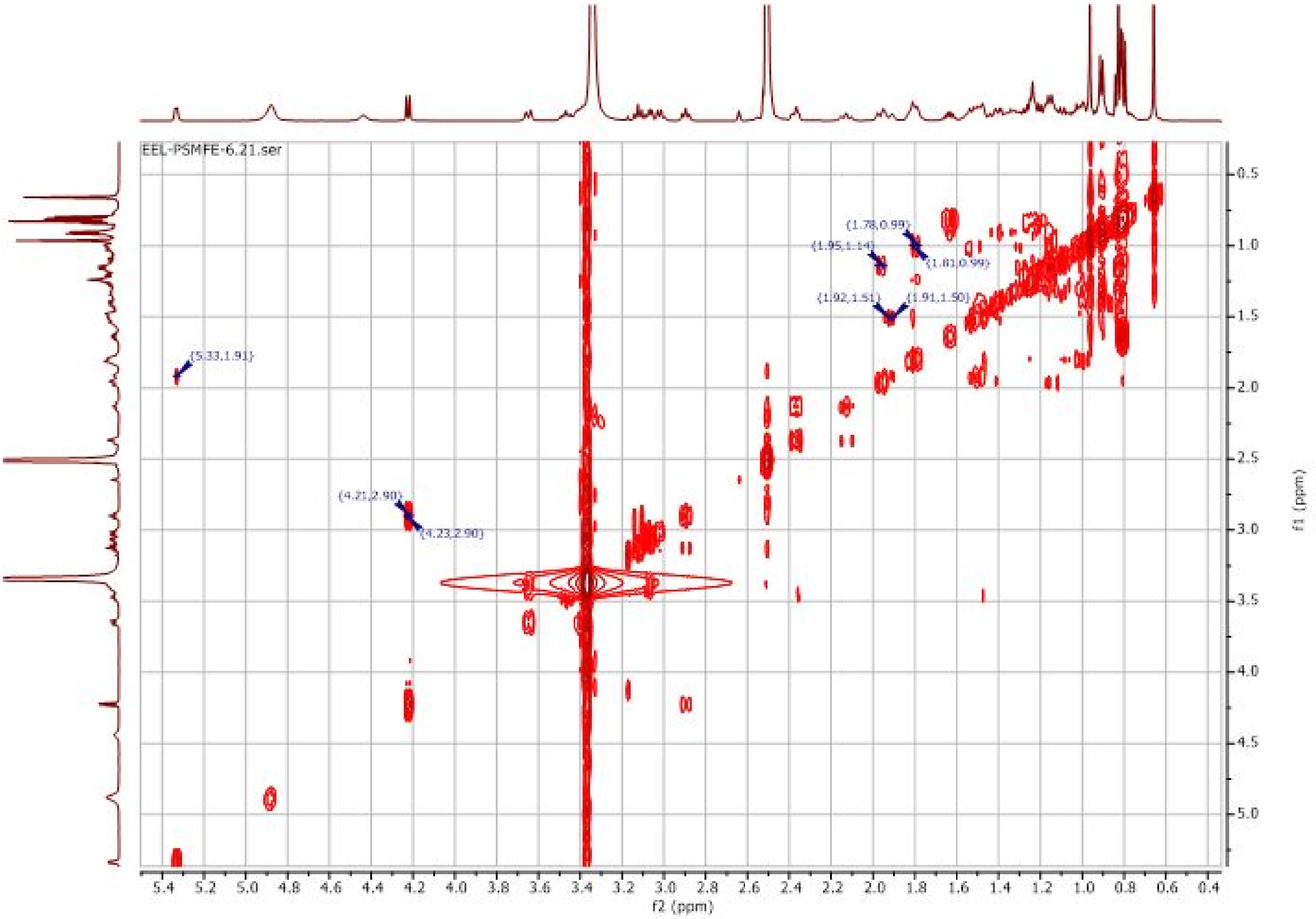

Figure S15: COSY spectrum of compound (**3**).

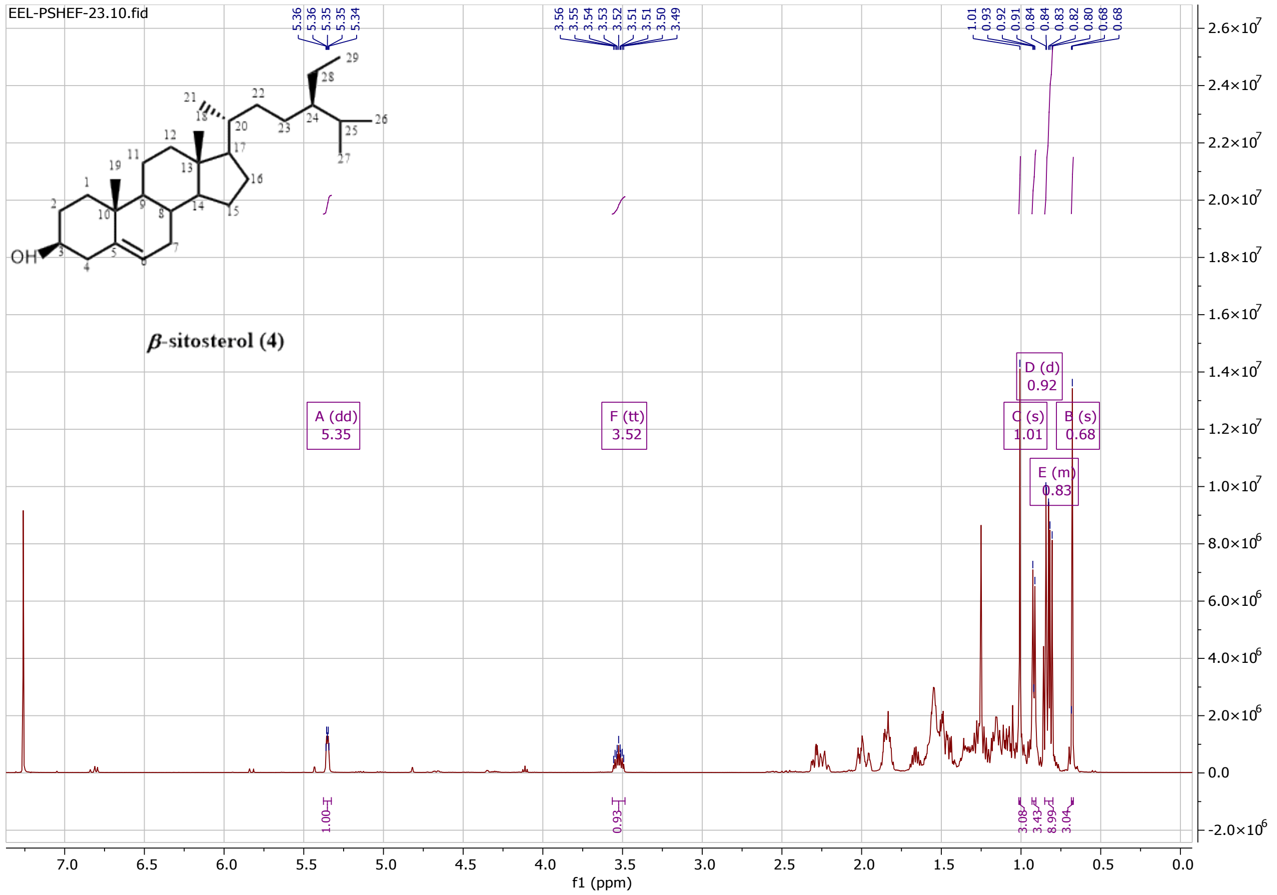

Figure S16: ^{1}H- NMR (500 MHz, $CDCl_3$) spectrum of compound (**4**).

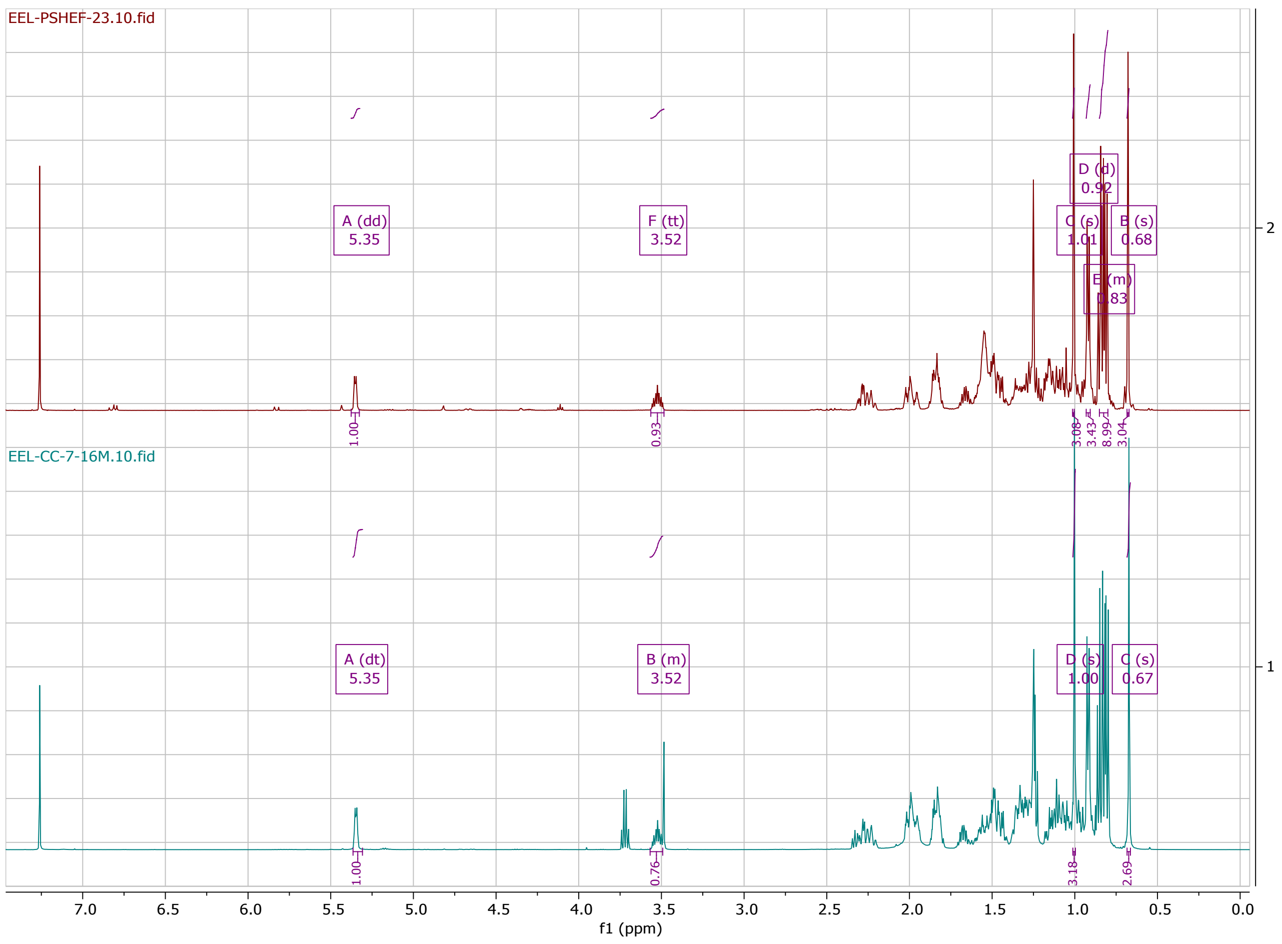

Figure S17: ^{1}H- NMR spectrum of compound (**4**) (top) in comparison to *β*-sitosterol isolated from *Caulerpa cylindracea* (bottom).

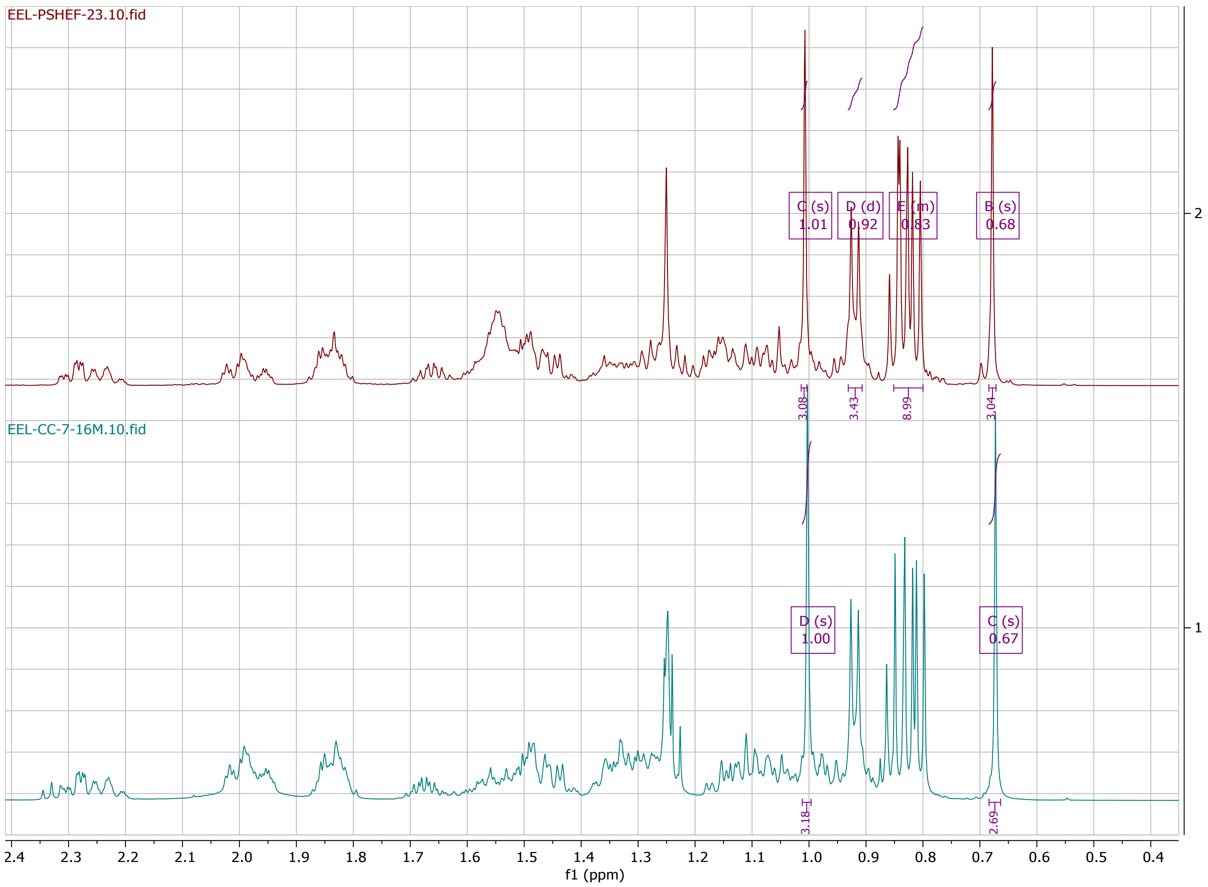

Figure S18: ^{1}H- NMR spectrum of compound (**4**) (top) in comparison to *β*-sitosterol isolated from *C. cylindracea* (bottom)-close-up view.

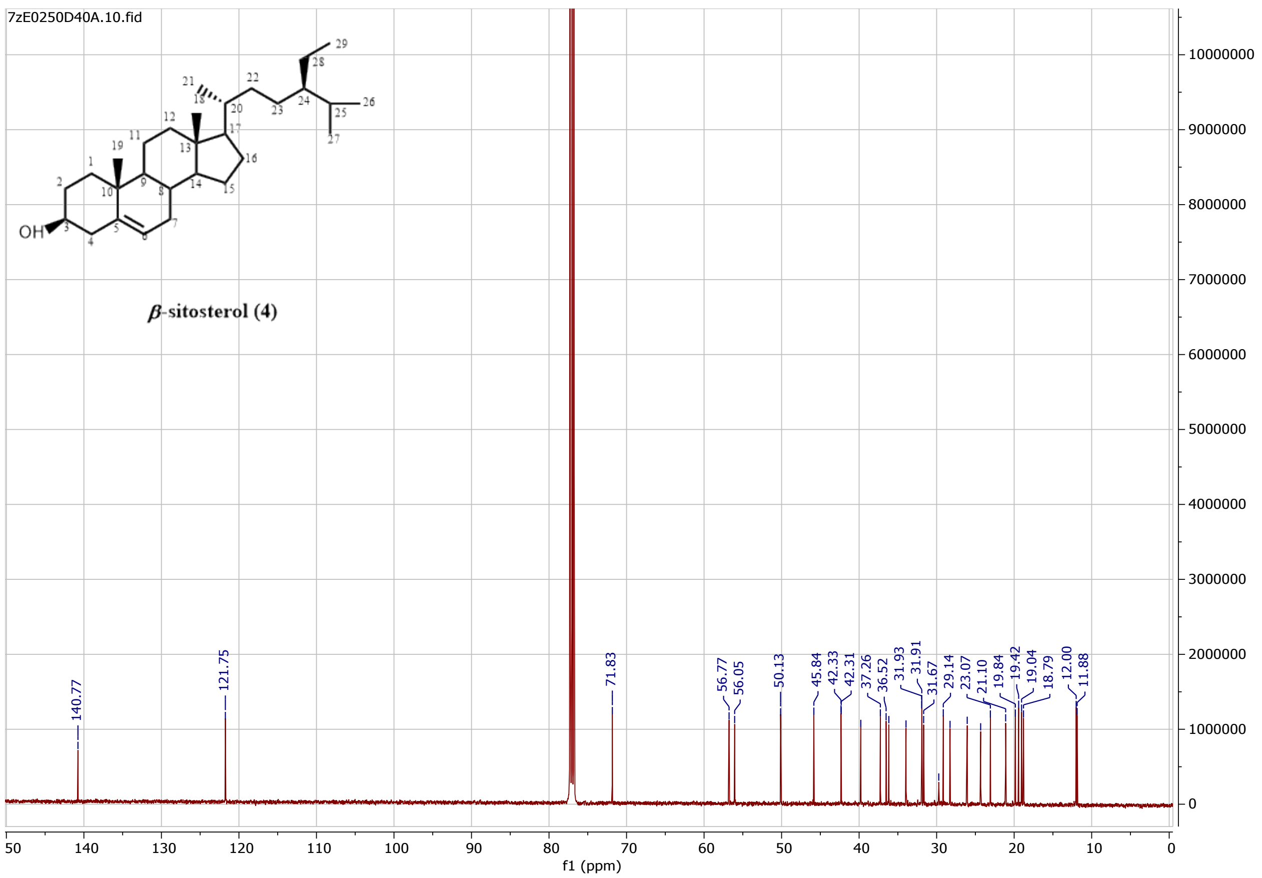

Figure S19: ^{13}C- NMR (125 MHz, $CDCl_3$) spectrum of compound (**4**).

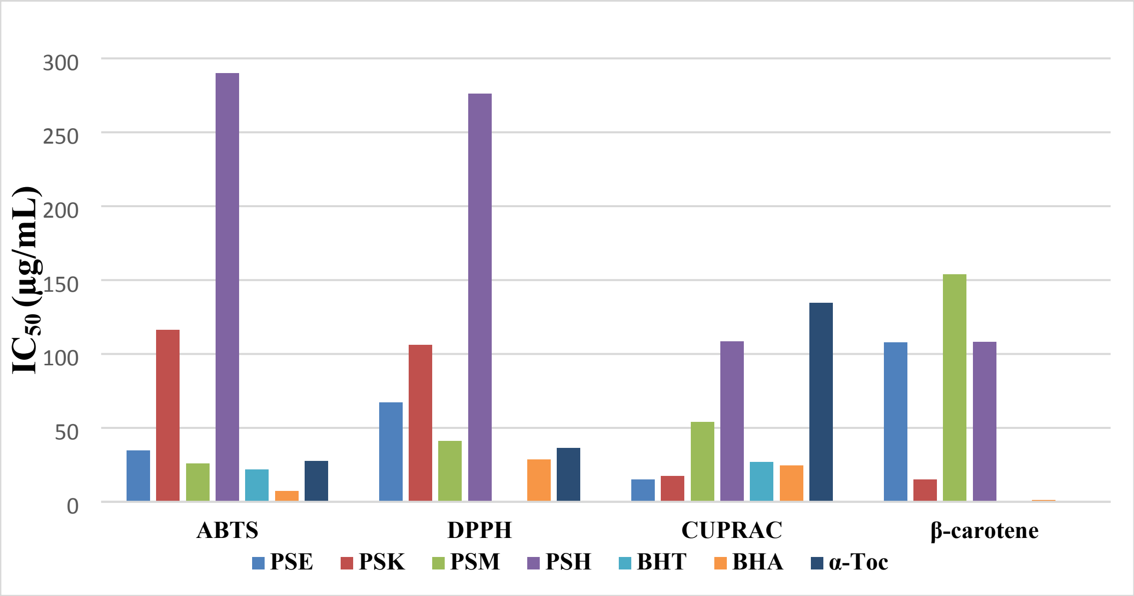

Figure S20. Antioxidant activity results of organic extracts in four different assays.

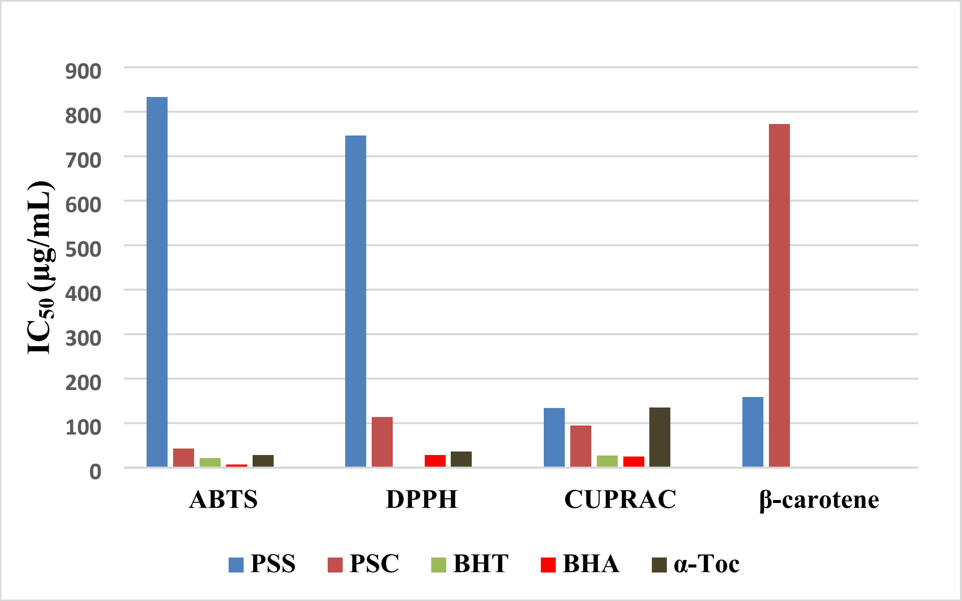

Figure S21. Antioxidant activity results of water and cooked extracts in four different assays.

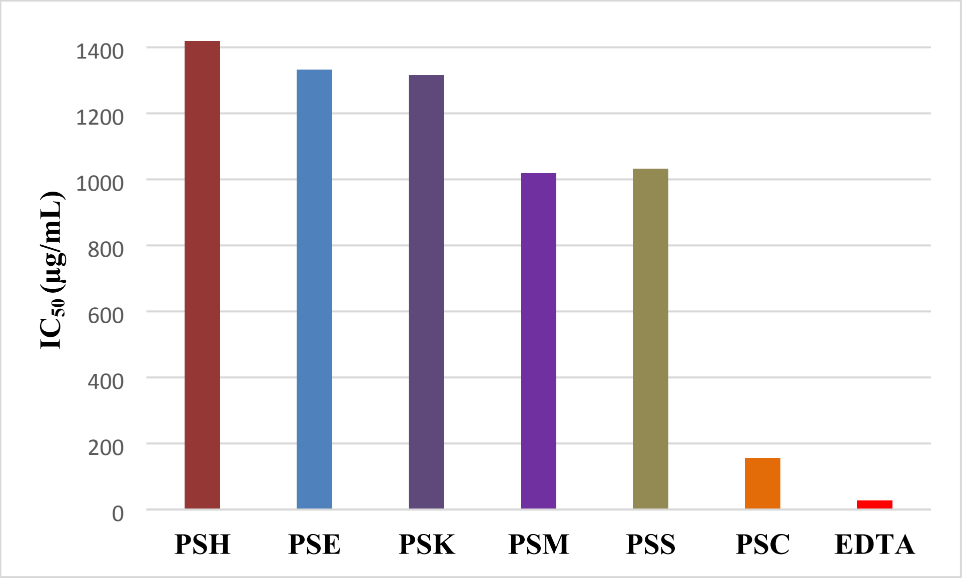

Figure S22. Metal chelating assay results of six different extracts and the standard.

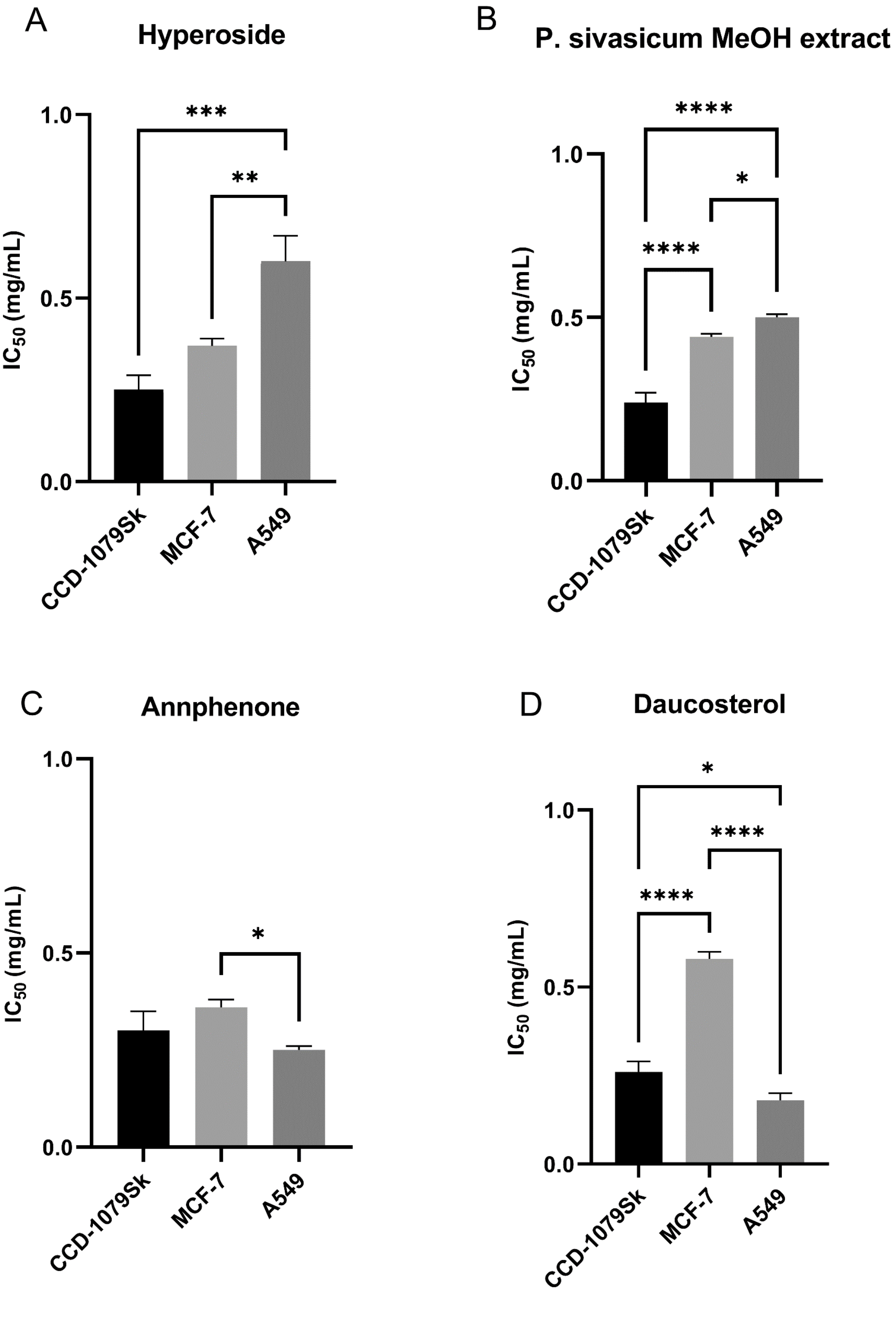

Figure S23. IC_{50} values of compounds on CCD-1079Sk, MCF-7 and A549 cells lines after 24 h of treatment. All the values are expressed with the mean±SD, n = 3. *$p < 0.05$, **$p < 0.01$, ***$p < 0.001$ and ****$p < 0.0001$
